# Engineered Pericyte‐Targeted Extracellular Vesicles Protect Against Hypoperfusion‐Induced Cognitive Impairment and Vascular Demyelination

**DOI:** 10.1002/jev2.70319

**Published:** 2026-06-01

**Authors:** Weiwei Shen, Weishi Liu, Min Guo, Tongyao You, Yingzhe Wang, Tiansiyu Wen, Suzhen Liang, Xiaodi Xie, Yanfeng Jiang, Qiang Dong, Jin‐Tai Yu, Mei Cui

**Affiliations:** ^1^ Department of Neurology and National Center for Neurological Disorders, Huashan Hospital Fudan University Shanghai China; ^2^ Human Phenome Institute, Zhangjiang Fudan International Innovation Center Fudan University Shanghai China; ^3^ State Key Laboratory of Brain Function and Disorders and MOE Frontiers Center for Brain Science, Shanghai Medical College Fudan University Shanghai China

**Keywords:** conjugation, demyelination, extracellular vesicles, pericyte

## Abstract

Pericyte dysfunction is an early hallmark of vascular cognitive impairment (VCI), yet targeted therapies remain limited. Here, we develop a bio‐orthogonal approach to engineer extracellular vesicles (EVs) functionalized with cyclic NGR (cNGR) peptides for selective pericytes targeting. The resulting cNGR‐EVs demonstrated efficient targeting of CD13‐expressing brain pericytes both *in vitro* and *in vivo*. In a mouse model of chronic cerebral hypoperfusion (bilateral common carotid artery stenosis, BCAS), cNGR‐EVs preserved pericyte contractility, protected against cerebral hypoperfusion, reduced blood‐brain barrier leakage, and attenuated demyelination, leading to improved cognitive performance. Single‐cell RNA sequencing further revealed that cNGR‐EVs attenuated the majority of BCAS‐induced transcriptional changes in pericytes, partially preserved pericyte‐mediated intercellular communication, and normalized downstream neuronal and glial gene expression profiles. These findings underscore cNGR‐EVs as a pericyte‐targeted strategy capable of stabilizing the neurovascular unit, maintaining pericyte function, and preventing cognitive decline and myelin loss, highlighting pericytes as a promising therapeutic target in early VCI treatment.

## Graphical abstract

Schematic illustration on a therapeutic strategy wherein engineered extracellular vesicles (EVs) are delivered intranasally and specifically target pericytes. In a model of chronic cerebral hypoperfusion (BCAS), this targeted approach preserves cerebral blood flow, reinforces blood‐brain barrier (BBB) integrity, stabilizes the neurovascular unit (NVU), mitigates demyelination, offering a promising therapeutic avenue for vascular cognitive impairment.

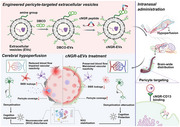

## 1 Introduction

Vascular cognitive impairment (VCI) encompasses a spectrum of cognitive disorders attributable to cerebrovascular pathology, ranging from mild cognitive impairment to vascular dementia. It represents the second leading cause of dementia worldwide, with prevalence increasing with age. Major risk factors include aging, hypertension, diabetes, dyslipidemia, and other vascular comorbidities. Clinically, VCI is typically characterized by prominent executive dysfunction and processing speed impairment, while memory deficits may occur depending on lesion distribution and concomitant neurodegenerative pathology. Current management primarily focuses on vascular risk factor control and secondary stroke prevention, with limited disease‐modifying therapies available. Emerging therapeutic strategies are increasingly targeting the underlying neurovascular and molecular mechanisms of VCI (Mok et al. 2024; Ng et al. 2025).

Chronic cerebral hypoperfusion serves as a core pathogenic mechanism of VCI (Ng et al. 2025). Despite this established link between cerebrovascular dysfunction, neurodegeneration and cognitive decline, the lack of effective interventions underscores a critical unmet need (Rundek et al. 2022). Pericytes have emerged as central players in VCI pathophysiology (Uemura et al. 2020). As specialized mural cells surrounding cerebral capillaries, pericytes dynamically regulate cerebral microcirculation through their contractile properties and maintain blood‐brain barrier (BBB) integrity (Armulik et al. 2010; Hartmann et al. 2022). Under chronic cerebral hypoperfusion, pericyte dysfunction precipitates a vicious cycle of microcirculatory failure and BBB breakdown, driving white matter injury, demyelination, and cognitive decline (Lin et al. 2024; Liu et al. 2019). Consequently, targeted interventions designed to preserve pericyte function offer a rational strategy to preserve cerebral perfusion, protect the neurovascular unit (NVU), and potentially modify the course of VCI.

However, targeted pericyte modulation remains constrained by two challenges: inefficient drug delivery across the endothelial cell and limited pericyte specificity (Cheng et al. 2018). The BBB excludes >98% of small‐molecule drugs, permitting passage only of lipophilic compounds under 500 Da or those exploiting active transport mechanisms (Alavijeh et al. 2005). Extracellular vesicles (EVs) have recently emerged as promising central nervous system (CNS) delivery vehicles owing to their innate biocompatibility, circulatory stability, and intrinsic BBB‐penetrating ability (You et al. 2024). These nanoscale lipid bilayers encapsulate functional proteins and nucleic acids derived from parent cells, traversing endothelial cells *via* membrane fusion or receptor‐mediated endocytosis, and potentially engaging pericytes during transit (Heidarzadeh et al. 2021). Upon internalization by recipient cells, the cargo delivered by EVs can induce functional and phenotypic changes (Gratpain et al. 2021). Bioengineering strategies further augment the therapeutic potential of EVs by enhancing production yield, biotherapeutic cargo loading and cell‐specific targeting (Teng and Fussenegger 2021). Harnessing the innate biological properties of EVs combined with advanced bioengineering approaches presents a transformative strategy for targeted modulation of pericyte dysfunction.

Brain‐targeted EVs engineering was pioneered by Matthew Wood's group through genetic modification of donor cells to express a rabies viral glycoprotein (RVG)‐Lamp2b fusion protein, enabling neuron‐directed siRNA delivery in Alzheimer's disease (AD) models (Alvarez‐Erviti et al. 2011). While genetic engineering facilitates the stable display of targeting ligands on EVs’ surface, its clinical translation faces challenges including complex manufacturing, cost constraints, and potential horizontal gene transfer risks (Pham et al. 2021). Post‐isolation surface functionalization offers a clinically viable alternative by leveraging a variety of reactive groups on EV membranes (–COOH, –NH_2_, –SH) for covalent conjugation (Asfiya et al. 2024). Click chemistry, particularly strain‐promoted azide‐alkyne cycloaddition (SPAAC), has emerged as a robust strategy (N'Diaye et al. 2022). NHS‐ester crosslinking enables the dibenzocyclooctyne (DBCO) group onto EVs membranes, subsequently reacting with azide‐modified targeting ligands (e.g., peptides, antibodies) (Richter et al. 2021). This modular approach achieves dual functionality: retention of vesicle structural integrity and programmable targeting specificity toward defined tissues or cell types (Gupta et al. 2021; Tian et al. 2018; Zhang et al. 2022).

Capitalizing on this strategy, we engineered disulfide‐cyclized CNGRC peptide (Asn‐Gly‐Arg, cNGR)‐functionalized EVs (cNGR‐EVs) *via* SPAAC for pericyte‐targeted delivery. The NGR motif, originally identified *via* phage display, binds specifically to CD13 isoforms. While extensively employed for tumor vasculature targeting due to its high affinity (Soudy et al. 2012), CD13 is also significantly enriched on cerebral pericytes under physiological conditions (Smyth et al. 2018). Crucially, single‐cell transcriptomics and FACS analyses have demonstrated that CD13 expression is more specific to pericytes than to other brain cell types, providing a precise molecular handle for targeted therapy (Crouch and Doetsch 2018; Vanlandewijck et al. 2018). To optimize this interaction, we utilized the disulfide‐cyclized variant, which confers superior structural stability and enhanced binding affinity to CD13 compared to its linear counterpart (Colombo et al. 2002). Upon binding, the NGR motif enhances nanoparticle‐cell membrane interactions, thereby facilitating the accumulation of therapeutic cargo within target cells.

Based on this, we evaluated whether cNGR‐EVs exhibit selective targeting towards CD13‐expressing pericytes. Following intranasal administration, cNGR‐EVs demonstrated significantly enhanced brain‐wide distribution and pericyte‐specific uptake across multiple regions compared to either unmodified EVs or scramble peptide modified EVs. To evaluate therapeutic potential, we employed the bilateral common carotid artery stenosis (BCAS) model–a well‐established surrogate of VCI (Che et al. 2023; Hattori et al. 2016). Intranasal delivery of cNGR‐EVs to BCAS mice improved vascular reactivity and cerebral blood flow (CBF), reduced BBB disruption, and attenuated demyelination, accompanied by improved cognitive performance. Furthermore, single‐nucleus RNA sequencing (snRNA‐seq) revealed that cNGR‐EVs mitigated BCAS‐induced alterations in the pericyte transcriptome, and were associated with partial normalization of neuronal and glial gene expression profiles. Collectively, these findings support cNGR‐EVs as a pericyte‐targeted delivery strategy with therapeutic potential for protecting neurovascular function in VCI.

## 2 Materials and Methods

### 2.1 Cell Culture

Human umbilical cord‐derived mesenchymal stem cells (hucMSCs) were obtained from the National Stem Cell Translational Resource Center and cultured in the provider's proprietary serum‐free medium. HT1080 (Anwei‐sci, #AW‐CH0155), HT29 (Anwei‐sci, #AW‐CH0157) and human brain vascular pericytes (HBVP; ZQXZ‐bio, #ZQY006), human primary cerebral vascular pericytes (PC; ZQXZ‐bio, #PRI‐H‐00170) were cultured in MEM (Gibco, #11095080) complete medium, McCoy's 5A (Anwei‐sci, #BasMed‐AW‐011) complete medium and specialty pericyte medium (ZQXZ‐bio, #ZMY006 and #ZQ‐1321) respectively. All cells were maintained at 37°C with 5% CO_2_ in a humidified incubator.

For EV uptake and functional assays, the complete media for HT29, HT 1080, HBVP and primary pericytes were initially cultured in the standard complete medium, which contains 2% FBS and defined growth supplements. To avoid interference from bovine EVs in functional assays, this medium was reconstituted using Exosome‐Free FBS (FUSHENBIO Co., FS0994‐50 mL). During EV treatment experiments, the culture medium was replaced with this EV‐depleted complete medium, and MSC‐derived EVs were added accordingly.

### 2.2 Isolation and Characterization of EVs

When hucMSCs reached ∼100% confluency in T75 flasks (10 mL medium), EVs were isolated from supernatants as described in our previous study (Shen et al. 2023). Briefly, supernatants were centrifuged at 400 × *g* for 5 min, 3000 × *g* for 15 min and 10,000 × *g* for 30 min at 4 °C to remove cell debris and microparticles. The supernatants passed through 0.45 µm‐syringe filters. Pellets were concentrated by using ultracentrifugation with a Type Ti70 rotor (Beckman Coulter, USA) at 120,000 × *g* for 90 min at 4 °C and were resuspended in phosphate‐buffered saline (PBS). The morphology of isolated EVs was analyzed by transmission electron microscopy (TEM) at the Chinese Academy of Sciences (see Section Section 2.12 for protocol). Particle concentration, size distribution and zeta potential were measured *via* nanoparticle tracking analysis (NTA) (ZetaView PMX 110, Particle Metrix) at Viva Cell Shanghai. Additionally, EV markers were detected *via* Western blot (see Section 2.13 for protocol).

### 2.3 Conjugation of EVs With Peptides/Fluorophores

Based on preliminary optimization, we established a SPAAC system to conjugate peptides and dyes to EVs. Briefly, MSC‐EVs (100 µg, ∼1.8 × 10^12^ EVs) were incubated with 100 µM DBCO‐sulfo‐NHS (18 mM stock, Sigma) for 3 h at room temperature (RT) under gentle rotation to introduce DBCO groups onto the EV surface. Unbound DBCO was removed by centrifugation (120,000 × *g*, 90 min, 4 °C), and EV pellets were resuspended in PBS.

For peptide functionalization, DBCO‐modified EVs were incubated with 10 µM azide‐modified peptides overnight at 4°C. Peptides included cNGR [(N3‐Acp)‐KCNGRC(S‐S)], scrambled control [(N3‐Acp)‐KCRNGC(S‐S)] and FITC‐labeled cNGR [(N3‐Acp)‐K(FITC)CNGRC(S‐S)] (synthesized by NJPeptide Co.). Excess peptides were removed by centrifugation (120,000 × g, 90 min, 4 °C).

For fluorescent labeling, peptide‐conjugated EVs were further incubated with 10 µM Cyanine3 azide (Cy3, Lumiprobe, #11030) or Cyanine7 azide (Cy7, Lumiprobe, #15030) overnight 4°C. In parallel, unmodified EVs were directly incubated with Cy3 or Cy7 azide under the same conditions to generate non‐targeted controls (Cy3/Cy7‐EVs). Free dye was removed using a 30% sucrose cushion followed by ultracentrifugation (120,000 × g, 90 min, 4 °C). The resulting pellets, including Cy3/Cy7‐cNGR‐EVs, Cy3/Cy7‐Scr‐EVs, and Cy3/Cy7‐EVs, were resuspended in PBS. Cy3‐labeled EVs were used for fluorescence imaging and uptake analyses. Cy7‐labeled EVs were used for near‐infrared fluorescence (NIRF) imaging.

### 2.4 Single‐EV Flow Cytometry

Single‐EV flow cytometry was performed on a CytoFLEX‐S flow cytometer (Beckman Coulter) following established protocols with modifications (Pham et al. 2021). Briefly, EVs were resolved from background using violet side scatter (VSSC; 405 nm laser). 100 nm polystyrene beads (Thermo Fisher Scientific) served as size reference. The system was pre‐rinsed with sterile distilled water (2 min, maximum flow rate) before it ran and between samples. Samples were analyzed at a 10 µL/min flow rate. Data collection commenced only after flow stabilization.

### 2.5 Atomic Force Microscopy (AFM)

Biomechanics of EVs were measured *via* AFM (SPM‐Nanoa, Shimadzu) at Beijing Zhongke Optical Analysis Chemical Technology Research Institute. The contact mode was performed using silicon probes (CSG30; spring constant: 0.6 N/m; tip radius: 10 nm; MDT&TipsNano). EVs were scanned at 5.0 Hz over 1.0 × 1.0 µm areas.

### 2.6 Flow Cytometry Analysis

To quantify CD13 expression, cells were harvested and incubated with an APC‐conjugated CD13 monoclonal antibody (Clone WM‐15, eBioscience, #17‐0138‐42) for 30 min at 4°C in the dark. Data were acquired on a CytoFLEX‐S cytometer (Beckman Coulter) and analyzed using CytExpert software. The pericyte population was identified by FSC/SSC gating and doublet exclusion, with CD13 expression quantified by the percentage of positive cells relative to negative controls.

To evaluate cNGR‐EVs targeting specificity *in vitro*, CD13‐positive (HT1080, HBVP) and CD13‐negative (HT29) cells were incubated for 2 h at 37°C with PBS or Cy3‐labeled EVs including Cy3‐EVs, Cy3‐Scr‐EVs and Cy3‐cNGR‐EVs. EV stock concentration (1 µg/ µL) was diluted to a working concentration of 45 µg/mL. Cells were trypsinized, washed, and sequentially processed with fixation buffer (30 min, RT) and permeabilization buffer (60 min, RT), with PBS washes (400 g, 5 min) between steps. Flow cytometry was performed on a CytoFLEX‐S cytometer (Beckman Coulter), with cells gated by FSC‐A/SSC‐A (viability), FSC‐W/FSC‐H (singlets), and PE channel (Cy3+ population). Data were analyzed using FlowJo v10 (FlowJo LLC).

### 2.7 EV Uptake Assays

HBVP/PC seeded on 13‐mm coverslips (24,000 cells/well) were incubated with 45 µg/mL Cy3‐labeled EVs (Cy3‐EVs, Cy3‐Scr‐EVs and Cy3‐cNGR‐EVs) for 2 h at 37°C, 5% CO_2_. Cells were washed with PBS, stained with CellMask DeepRed (1×, 10 min, 37°C; Thermo Fisher), fixed with 4% paraformaldehyde (15 min, RT), and mounted with DAPI (Southern Biotech). Imaging was performed using an Olympus SpinSR confocal microscope (60×, 100× oil objective). For semi‐quantification, five random fields per specimen were analyzed in ImageJ (v1.8.0, NIH) by measuring mean Cy3 pixel intensity within ROIs defined by CellMask. For the removal of terminal sialic acids and O‐glycans, PC were pre‐treated with 0.5 U/mL neuraminidase (NA; Sigma‐Aldrich, #N6514) for 1 h at 37°C. Following treatment, cells were washed twice with PBS to remove residual enzyme before being incubated with MSC‐derived EVs in fresh culture medium.

### 2.8 Animals and Study Design

Male C57BL/6J mice (12‐week‐old, 25–30 g; Charles River Laboratories) were housed in the Fudan University Experimental specific pathogen‐free animal facility. All procedures complied with the National Science Council of China guidelines and were approved by the Fudan University Ethics Committee (IRB approval number: 2022‐JSHSYY‐283). Age‐matched mice were randomly allocated to experimental groups and data were analyzed blindly. Separate cohorts of mice were used for each experimental endpoint to avoid confounding effects. Sample sizes (*n*) were consistent with standard practices in the field. All animal studies adhered to ARRIVE guidelines.

Biodistribution of cNGR‐EVs was assessed in normal mice to establish baseline pericyte‐targeting capability. Functional and therapeutic evaluations were conducted in BCAS mice with sham‐operated controls, as described below. Vascular function (e.g., pericyte coverage, capillary reactivity) was assessed 3 days after EVs administration. Blood flow was monitored at 3, 7, and 30 days post‐administration, whereas pathological outcomes (demyelination), behavioral performance, and transcriptomic analyses were evaluated at 30 days post‐treatment.

### 2.9 EVs Biodistribution in Vivo

Mice were intranasally administered with PBS or Cy3‐labeled EVs (Cy3‐EVs, Cy3‐Scr‐EVs and Cy3‐cNGR‐EVs), followed by sacrifice 2 h post‐delivery. Following anesthesia and transcardial perfusion, brains were post‐fixed in 4% PFA overnight, dehydrated in 20%/30% sucrose gradients, and embedded in O.C.T. compound (Tissue‐Tek) for cryosectioning. Serial 30‐ µm sagittal sections were collected using a Leica cryostat. For pericyte identification, sections were immunostained with anti‐PDGFR‐β (1:100, Abcam #32570) and appropriate fluorescent secondary antibodies (Thermo Fisher). Confocal imaging was performed on an Olympus SpinSR system (60×, 100× oil objective) with z‐stack acquisition. Quantitative analysis of EVs incorporation was conducted in the olfactory bulb (OB), cortex (Ctx), corpus callosum (CC), and other subcortical structures (SCtx) including striatum (Str), hippocampus (Hip), pons (Pn), and cerebellum (Cb).

### 2.10 BCAS Model and Intranasal Administration of Evs

The BCAS procedure was performed as previously described with modifications (Feng et al. 2021). Mice were anesthetized with 4% isoflurane (28% O_2_/68% N_2_) and maintained at 2% isoflurane (29% O_2_/69% N_2_) *via* facemask. Following neck midline incision, bilateral common carotid arteries were exposed and stenosed using 0.18‐mm‐diameter steel microcoils (Wuxi Samini). Sham controls underwent identical procedures without coil placement. Postoperative intranasal EVs administration (unmodified or cNGR‐modified) was initiated upon recovery from anesthesia.

Intranasal administration of EVs was performed immediately after recovery from anesthesia, with a single dose delivered per mouse, as previously described (Shen et al. 2023). Briefly, mice were pretreated bilaterally with 10 µL hyaluronidase (100 U in PBS; Sangon Biotech) per nostril, delivered in ∼2 µL droplets at the nostril entrance and drawn in by the mouse's natural inhalation. Droplets were alternated between nostrils with a 2‐min interval until the full volume was delivered. After 30 min, 50 µL cNGR‐EVs, control EVs (100 µg), or PBS was administered intranasally. EVs were administered as sequential 2 µL droplets (10 µL micropipette), alternating between nostrils every 2 min to allow spontaneous inhalation until the full dose was delivered.

### 2.11 Behavioral Experiment

Eight‐arm radial maze: Spatial memory was assessed using an eight‐arm radial maze (Auto‐8ArmMaze, Shanghai Vanbi Intelligent Technology Co., Ltd). Mice were habituated to the maze during a 10‐min pretraining session with scattered food pellets on days 1 and 2. On days 3 and 4, mice were trained to retrieve food pellets from arm‐end containers, with two training sessions per day. For testing, four arms were pseudorandomly baited. After a 30‐sec delay on the central platform, mice were allowed to freely explore the maze for up to 10 min. Testing was conducted twice daily from day 5 for 7 consecutive days.Working memory errors (re‐entries into baited or unbaited arms) and reference memory errors (entries into never‐baited arms) were recorded. Trials were video tracked by Tracking Master (v5.0), and the maze was cleaned with 70% ethanol between sessions.

Elevated plus maze (EPM): Anxiety‐like behavior was evaluated using EPM, consisting of two open arms (30 cm × 5 cm) and two enclosed arms (30 cm × 5 cm × 15 cm) extending from a central platform (5 × 5 cm), elevated 90 cm above the ground. Mice were placed individually in the center, and their activity was recorded for 5 min *via* EthoVision XT (Noldus, v14.0). The number of entries into and time spent in each arm were quantified. Anxiety‐related behavior was expressed as the percentage of open arm entries (%OAE = [open/total entries] × 100) and percentage of time spent in open arms (%OT = [open/total time] × 100). The apparatus was cleaned with 70% ethanol between trials to eliminate residual odors.

### 2.12 Tem

EV samples: EV samples were diluted in PBS and applied to carbon‐coated copper grids (3∼5 µL). Grids were briefly rinsed with distilled water, stained with 1% uranyl acetate, and air‐dried. Imaging was performed using a JEOL 1230 TEM at 80 kV. Digital micrographs were acquired with a Gatan Orius 830 CCD camera (2048 × 2048 pixels).

Brain samples: Mice were transcardially perfused with 0.1 M PBS, followed by a fixative solution of 4% paraformaldehyde and 1% glutaraldehyde in 0.1 M phosphate buffer. Brains were dissected and post‐fixed in 2.5% glutaraldehyde (3 h, 4°C). Coronal sections (150 µm) were prepared using a vibratome (Leica), andCC fragments were isolated under a stereomicroscope. For TEM, samples were processed as previously described. Ultrathin sections (70 nm) were mounted on coated copper grids and examined using a JEOL 1230 TEM (Chinese Academy of Sciences). Myelin integrity was assessed by quantifying fibers with loosely wrapped lamellae or interlamellar cavities, classified as “demyelinated.” Myelinated axon counts and G‐ratios (inner‐to‐outer axonal circumference) were analyzed using ImageJ software.

### 2.13 Western Blot Analysis

For CCprotein analysis, mice were deeply anesthetized with 5% isoflurane in air and transcardially perfused with ice‐cold PBS. The whole brain was rapidly removed and placed into ice‐cold PBS. Coronal slices of 500 µm thickness were prepared using a vibrating microtome. Under a stereomicroscope, the CC was carefully dissected from the overlying gray matter (cortex) and subcortical structures (e.g., thalamus) using straight and curved forceps. The CC is readily identifiable by its white matter appearance and can be traced along its anatomical course. Dissected CC tissue was immediately snap‐frozen in liquid nitrogen and stored at −80°C until further processing.

EVs, cell, and CC lysates were prepared using RIPA buffer supplemented with protease inhibitors. Protein concentrations were determined *via* BCA assay (Sangon Biotech). Equal amounts of protein (20 µg for EV/cell lysates; 30 µg for CC tissue) were resolved by SDS‐PAGE (concentration optimized for target molecular weights) and transferred to 0.22 µm PVDF membranes. After blocking with 5% skim milk, membranes were incubated overnight at 4°C with primary antibodies against: Alix (1:1000, Abcam #117600), TSG101 (1:1000, Abcam #125011), CD63 (1:1000, Abcam #271286), Calnexin (1:1000, Abcam #22595), GM130 (1:500, BD Transduction Laboratories #610822), CD13 (1:1000, Abcam #108310), myelin basic protein (MBP; 1:1000, Abcam #218011), neurofilament (1:500, Abcam #7794), and ɑ‐tubulin (1:20,000, Proteintech #66031‐1‐Ig; loading control). Following three 10‐min TBST washes, membranes were incubated with HRP‐conjugated secondary antibodies (90 min, RT) and visualized using a ChemiDoc XRS+ System (Bio‐Rad).

### 2.14 Laser Speckle Blood Flow Imaging

Mice were anesthetized with 4% isoflurane (28% O_2_/68% N_2_) and maintained at 2% isoflurane (29% O_2_/69% N_2_) *via* facemask. Following scalp disinfection, a midline incision was made, and the skull surface was moistened with saline. Eyes were protected with erythromycin ointment, and the skull was covered with saline to maintain a thin hydration layer. Laser speckle imaging commenced when vascular morphology was clearly visible, and blood flow stabilized. Two consecutive recordings were obtained at 5‐min intervals. Post‐procedure, mice recovered on a warming pad. Cerebral blood flow was measured at baseline (set as 100%) and at 3, 7, and 30 days post‐BCAS surgery, with subsequent values normalized to baseline.

### 2.15 Pericyte Coverage

Mice were anesthetized and transcardially perfused, followed by tissue dehydration 3 days post‐surgery. Serial 30‐ µm coronal sections were obtained using a Leica cryostat. Immunofluorescence staining was performed using primary antibodies anti‐PDGFRβ (1:100, Abcam #32570) and anti‐CD31 (1:50, BD Pharmingen #550274), followed by appropriate fluorescent secondary antibodies. Images were obtained by an Olympus SpinSR confocal microscope (40× air objective). Pericyte coverage was quantified as the percentage of PDGFRβ‐positive area colocalized with the CD31 signal using ImageJ software.

### 2.16 Imaging Pericytes and Capillary Reactivity in Brain Slices

Mice were deeply anesthetized with 2% isoflurane and decapitated. Brains were rapidly extracted and immersed in ice‐cold artificial cerebrospinal fluid (ACSF) continuously aerated with 5% CO_2_ and 95% O_2_. Coronal slices (150 µm) were prepared using a Leica vibratome maintained in ice‐cold oxygenated ACSF. Slices were gently transferred to glass‐bottom confocal dishes containing 3 mL oxygenated ACSF and stabilized with a slice anchor. Capillaries were identified as healthy based on three criteria: (1) diameter <10 µm, (2) patent lumen containing biconcave erythrocytes, and (3) characteristic “bump‐on‐a‐log” pericyte somata (Nortley et al. 2019). Time‐series differential interference contrast (DIC) imaging was performed using an Olympus SpinSR confocal microscope (100× oil objective, NA 1.4) with the following timeline: 5 min baseline recording (pre‐drug), 15 min during 2 µM noradrenaline (NA) superfusion, 10 min during co‐application of 2 µM NA + 500 µM glutamate and 5 min washout period. For each mouse, at least 3 brain slices were analyzed, with at least 3 healthy capillaries imaged per slice. Luminal diameters were measured at the point of maximal response using OlyVIA software (v2.9.1), with vessel boundaries manually traced using the line‐drawing function across the entire time series.

### 2.17 Analysis of snRNA‐seq Data

Cell clustering and identification: Cell clustering was performed using the FindClusters function in Seurat (resolution = 1.0) (Hao et al. 2024), and cellular distribution was visualized in two‐dimensional space using Uniform Manifold Approximation and Projection (UMAP). Through unsupervised clustering, cells were categorized into 55 distinct clusters. Based on existing literature and the CellMarker database (https://xteam.xbio.top/CellMarker/), marker genes for relevant cell types were identified, enabling annotation of the clusters, classification of distinct cell types, and assessment of their distributions and proportions (Chen et al. 2017; Hajdarovic et al. 2022). To further investigate pericyte heterogeneity, these cells were isolated and subjected to independent re‐clustering, with each subpopulation annotated according to its unique gene expression signature.

Analysis of pericyte subpopulations: Pseudotemporal trajectories of pericyte subpopulations were inferred using Monocle2 (v2.22.0) with the DDRTree algorithm (Qiu et al. 2017), ordering cells along pseudotime to delineate developmental progression. Cell‐cell communication analysis was conducted with the R package CellChat (v2.12) under default settings, employing the CellChatDB mouse database (Jin et al. 2025). To quantify intercellular communication alterations, we used CellChat to calculate differences in communication strength for three pairwise comparisons: Sham versus BCAS, BCAS + EVs versus BCAS, and BCAS + cNGR‐EVs versus BCAS. The resulting communication strength differences were *z*‐score normalized for each comparison, and the normalized data were visualized in a heatmap.

Enrichment analysis of differentially expressed genes (DEGs):DEGs across pericyte subpopulations were identified with the FindAllMarkers function, employing a Wilcoxon rank‐sum test under default parameters. For comparative analysis among the three samples, the FindMarkers function was applied. Genes exhibiting a log2 fold change (log2FC) ≥1 and a *p*‐value < 0.05 were considered statistically significant and defined as DEGs. To elucidate the functional attributes of each cell subpopulation, Gene Ontology (GO) and Kyoto Encyclopedia of Genes and Genomes (KEGG) pathway enrichment analyses were performed on the DEGs using the clusterProfiler package (Yu et al. 2012). The resulting enrichment outcomes were visualized with ggplot2. Nine‐quadrant plots were generated using the results of differential gene expression analysis for the BCAS versus Sham comparison, paired with either BCAS + EVs versus BCAS or BCAS + cNGR‐EVs versus BCAS. Colored points in quadrants Q1 (Q1_sig) and Q9 (Q9_sig) represent genes with |fold change (FC)| > 2 and *p* value < 0.05 in both comparison groups. The proportion of Q1_sig genes was calculated as (Q1_sig / (Q1 + Q4 + Q7)), and the proportion of Q9_sig genes was calculated as (Q9_sig / (Q3 + Q6 + Q9)).

### 2.18 Statistical Analysis

Data are expressed as mean ± SD as listed in figure legends. All analyses were performed with GraphPad Prism software. Our sample sizes are similar to those reported in previous publications (Pham et al. 2021; Sun et al. 2020; Tian et al. 2018). The values of ‘*n*’ (sample size) are provided in the figure legends and supplementary source data. Normality was verified using the Shapiro–Wilk test, while homogeneity of variance was assessed using the Brown–Forsythe test. For normally distributed data with equal variances, statistical significance was determined by one‐way ANOVA or a Mixed‐effects model (REML) followed by Tukey's multiple comparisons test, depending on the experimental design. In cases where the assumption of equal variances was violated, Welch's ANOVA with Dunnett's T3 multiple comparisons test was used. For non‐normally distributed data, the Kruskal–Wallis test followed by Dunn's multiple comparison test was applied. The null hypothesis was rejected when the *p*‐value was <0.05. A detailed quantification workflow was described in the Supplementary Methods, attached with a Sample Size Table.

## 3 Results

### 3.1 Construction and Characterization of cNGR‐Functionalized EVs

**FIGURE 1:**
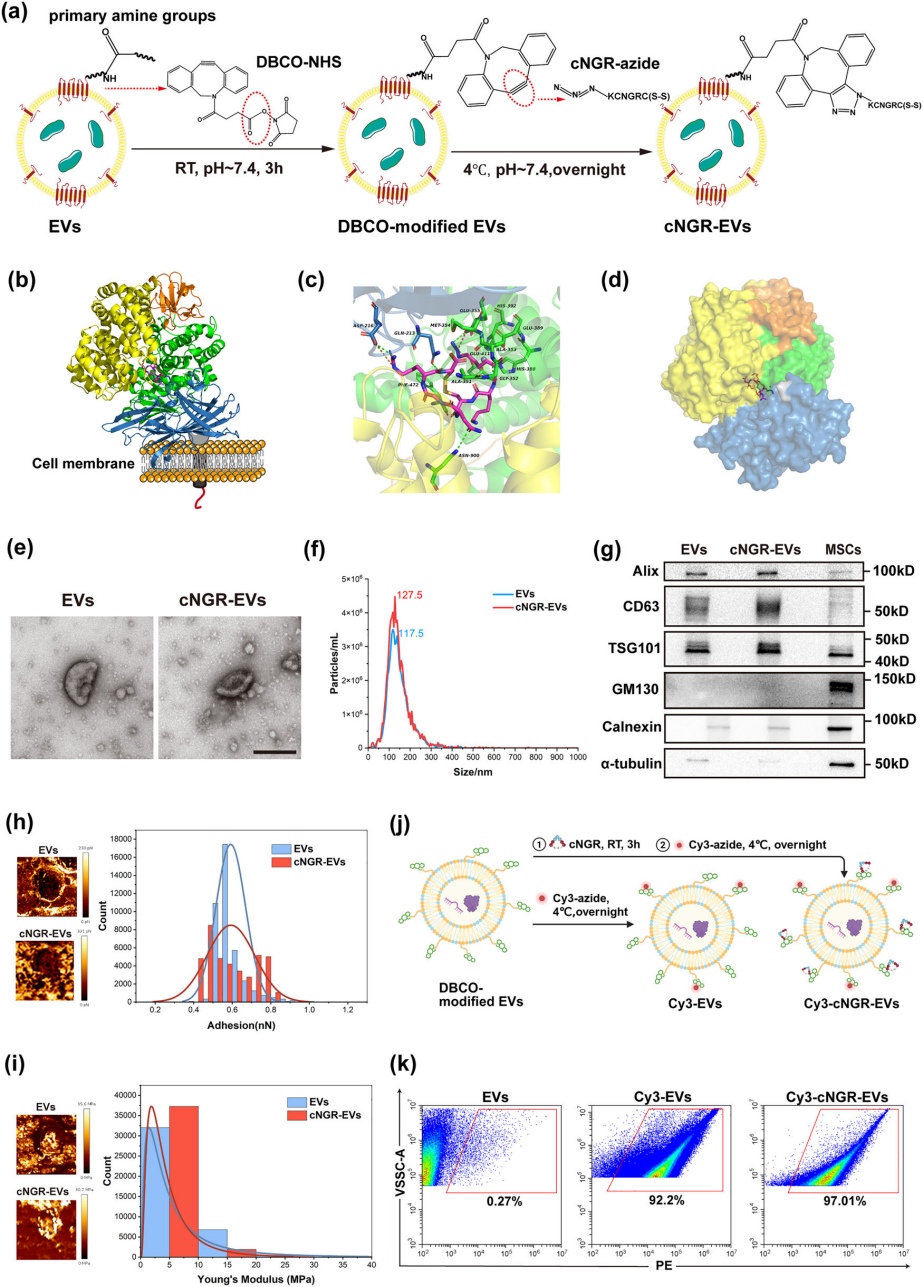
**Modification and characterization of cNGR‐functionalized EVs. (a)** Schematic illustration of cNGR peptide conjugation onto the EVs surface *via* SPAAC. EVs were first functionalized with DBCO groups through amide bond formation, followed by covalent conjugation with azide‐modified cNGR peptides to generate cNGR‐EVs. **(b)** Structure model of the human CD13 (hCD13)‐cNGR peptide complex. The hCD13 is depicted with its ectodomain (ribbon representation), a stalk (gray sphere), a transmembrane anchor (black cylinder), and an intracellular tail (red line). The ectodomain contains four domains: I (blue), II (green), III (orange), and IV (yellow). The cNGR peptide is shown as magenta sticks representation. One monomer of the dimeric hCD13 is displayed. **(c)** Close‐up view of the cNGR binding pocket. cNGR (magenta sticks) interacts with residues from hCD13 domains. Hydrogen bonds and salt bridges/charge interactions are indicated by green and orange dashed lines, respectively. The catalytic Zn^2+^ ion is shown as a gray sphere. **(d)** Surface representation of the hCD13–cNGR complex, highlighting the binding pocket accommodating the cNGR peptide. The hCD13 molecular surface is rendered semi‐transparent (domains colored as in b) to visualize the bound cNGR peptide (magenta sticks) within its binding pocket. **(e)** Transmission electron microscopy (TEM) images of negative control EVs (EVs) and cNGR‐EVs. Scale bar: 100 nm. **(f)** Representative nanoparticle tracking analysis (NTA) size distribution profiles of EVs and cNGR‐EVs. **(g)** Western blot analysis of EVs markers (Alix, CD63, TSG101) and negative controls (GM130, calnexin, α‐tubulin) in purified EVs, cNGR‐EVs, and parental cell lysates. **(h‐i)** Atomic force microscopy (AFM) analysis of nanomechanical properties of EVs and cNGR‐EVs: (**h**) Adhesion force maps and distribution histograms; **(i)** elasticity (Young's modulus) maps and distribution. **(j)** Schematic of EVs labeling strategy. DBCO‐modified EVs were sequentially conjugated with cNGR‐azide and Cy3‐azide to generate Cy3‐cNGR‐EVs. Cy3‐labeled EVs without cNGR modification (Cy3‐EVs) were used as a non‐targeted control. **(k)** Single‐EV flow cytometry analysis of labeling efficiency for EVs (control EVs without any modification), Cy3‐EVs and Cy3‐cNGR‐EVs. 100‐nm beads were used as size reference.

To functionalize EVs with cNGR, we employed a two‐step conjugation approach adapted from a previously reported method (Tian et al. 2018) (Figure 1a). In the first step, DBCO groups were introduced onto the EVs surface *via* reaction of DBCO‐NHS ester with primary amines present on the EVs membrane. This was followed by conjugation of azide‐modified cNGR to DBCO‐modified EVs *via* SPAAC, forming stable triazole crosslinks. Molecular docking simulations were performed to characterize the interaction between the cNGR peptide and the human CD13 (hCD13) receptor (Figure 1b‐d, Figure S1a). The docking score of cNGR binding to CD13 was ‐23.67 kcal/mol, suggesting favorable binding. cNGR exhibits complementary steric matching within the CD13 binding pocket and key interactions stabilized the complex (Figure 1c, Figure S1a), which may contribute to the predicted binding affinity. Based on titration results, a concentration of 10 µM cNGR was selected for conjugation with 100 µg DBCO‐modified EVs, achieving approximately 90% saturation while minimizing excess peptide usage (Figure S1b‐d). It was estimated that approximately 1.8 × 10^3^ FITC‐cNGR molecules were conjugated per EV (Figure S1e).

TEM analysis revealed that cNGR‐EVs retained a morphology similar to that of negative control EVs, suggesting that the conjugation process did not compromise vesicle structure or integrity (Figure 1e). NTA demonstrated a size distribution of 50‐200 nm for both control EVs and cNGR‐EVs, with a slight increase in diameter observed after cNGR modification (Figure 1f). The mean particle‐to‐protein ratio was approximately 3.28 × 10^8^ particles/ µg protein, which is consistent with reported values (Webber and Clayton 2013, Yang et al. 2024), under similar preparation and indicates acceptable purity. Western blot analysis confirmed the enrichment of EV markers, including both transmembrane (CD63) and cytosolic markers (Alix and TSG101) in EVs isolates relative to parental cell lysate. In contrast, negative markers such as GM130 (Golgi marker), calnexin (ER marker) and α‐tubulin (possibly non‐EV co‐isolated structures) were largely absent in EVs preparations (Figure 1g), supporting minimal contamination.

AFM analysis was performed to characterize the nanoscale biomechanical properties of EVs, focusing on elasticity (quantified by Young's modulus) and adhesion forces, both of which are known to influence EVs‐cell interactions (Zamith‐Miranda et al. 2021). Compared with control EVs, cNGR‐EVs moderately increased adhesion forces (Figure 1h) and elasticity (Figure 1i). These changes may contribute to enhanced interactions with recipient cells. To assess whether cNGR conjugation affected colloidal stability, the size of control EVs and cNGR‐EVs was measured on day 1 and day 3 under storage conditions (4°C in PBS). Both control EVs and cNGR‐EVs remained stable over time (Figure S1f).

For tracking experiments, EVs were labeled with Cy3 *via* azide conjugation (Figure 1j). Labeling efficiency was confirmed by single‐EV flow cytometry, demonstrating a high labeling rate (Figure 1k). Notebly, Cy3 labeling did not alter the particle size of either cNGR‐EVs or control EVs (Figure S1g). Therefore, Cy3‐labeled vesicles were used exclusively for uptake experiments (both in vitro and in vivo) and not for functional assays.

### 3.2 CNGR Functionalization Enhances CD13 Targeted Uptake and Preserves Pericyte Function Under Hypoxia

**FIGURE 2:**
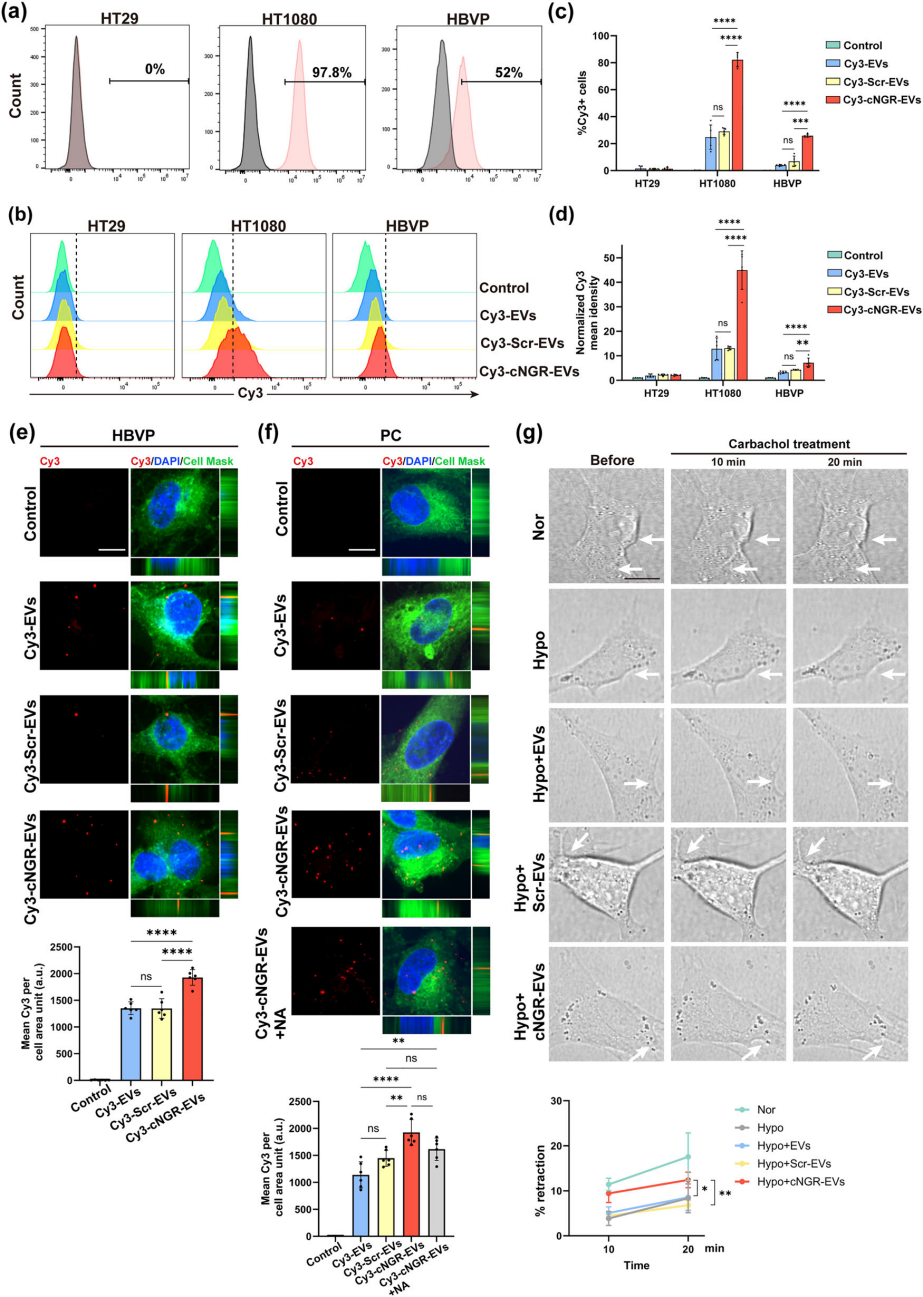
**Targeting specificity of cNGR‐EVs to CD13‐positive cells and its impact on pericyte contractility under hypoxic stress**. (a) CD13 isoform expression in HT29, HT1080 and HBVP quantified by flow cytometry. (b‐d) Cellular uptake of Cy3‐labeled EVs variants (Cy3‐EVs, Cy3‐Scr‐EVs, Cy3‐cNGR‐EVs) in HT29, HT1080 and HBVP, analyzed by flow cytometry. Quantitative analysis of the percentage of Cy3^+^ cells (c) and intracellular Cy3 fluorescence intensity (d) after 2 h of incubation. *n* = 5 independent experiments. (e) Confocal microscopy images of HBVP cells (CellMask‐stained, green) showing internalization of Cy3‐labeled EV variant (red) with *z*‐stack acquisition and orthogonal view. Quantification of mean Cy3 fluorescence intensity per cell area is shown. Scale bar: 10 µm. *n* = 6 independent experiments. (f) Confocal microscopy of primary pericytes (PC, CellMask‐stained, green) showing internalization of Cy3‐labeled EV variants (red) with *z*‐stack acquisition and orthogonal view. Quantification of mean Cy3 fluorescence intensity per cell area is shown. Scale bar: 10 µm. *n* = 6 independent experiments. (g) Carbachol‐induced contractility of primary pericytes. Representative images show morphological changes before and at 10 and 20 min after stimulation. Quantification of cell area contraction is shown. Scale bar: 10 µm. *n* = 6 independent experiments. Data are presented as mean ± SD. Detailed statistical information is provided in the source data Table S1. *P < 0.05, **P < 0.01, ***P < 0.001, ****P < 0.0001. Abbreviations: HBVP, human brain vascular pericytes; PC, human primary cerebral vascular pericytes; NA, neuraminidase; Nor, normoxia; Hypo, hypoxia.

To assess the targeting specificity of cNGR‐EVs, scrambled peptide‐conjugated EVs (Scr‑EVs) as a size‐matched non‐targeting control to exclude size‐dependent effects. Scr‐EVs were verified to possess similar structural characteristic, size distribution and Cy3 labeling efficiency to cNGR‐EVs (Figure S2a‐c). We first evaluated CD13 expression in established cell lines: CD13‐negative HT29, CD13‐positive HT1080, and human brain vascular pericytes (HBVP), the latter of which express high levels of CD13 and represent a relevant CNS target (Enyedi et al. 2017; King et al. 2024). Flow cytometry using the WM15 antibody which recognizes the CD13 isoform associated with NGR binding, showed that HT29 was CD13‐negative, HT1080 was ∼97% positive, and HBVP was ∼52% positive (Figure 2a). Consistent with this, CD13 protein levels were substantially lower in HBVP than in HT1080, with minimal expression in HT29 (Figure S2d).

Cells were incubated for 2 h with Cy3‐labeled EVs variants, including Cy3‐EVs, Cy3‐Scr‐EVs, and Cy3‐cNGR‐EVs.The percentage of Cy3+ cells and Cy3 fluorescence intensity were assessed by flow cytometry (Figure 2b). Quantification of Cy3^+^ cell populations confirmed significant increase in labeled HT1080 and HBVP cells following Cy3‐cNGR‐EVs treatment, whereas no difference was observed in CD13‐negtive HT29 cells (Figure 2c). In CD13‐negtive HT29 cells, all EV variants showed comparable association levels (Figure 2d). In contrast, CD13^+^ HT1080 cells exhibited approximately ∼3.5‐fold higher association of Cy3‐cNGR‐EVs relative to Cy3‐Scr‐EVs or Cy3‐EVs, while HBVP cells showed a similar but less pronounced trend, consistent with their lower CD13 expression (Figure 2d). Immunofluorescence analysis of HBVP cells showed stronger fluorescence signals after Cy3‐cNGR‐EVs incubation, further supporting the flow cytometry results (Figure 2e).

Having established preferential association of cNGR‐EVs with CD13‐expressing cells, we next examined their functional effects on HBVP cells *in vitro*. CCK8 assays showed no significant difference in viability across treatment groups (Figure S2e), indicating minimal cytotoxicity. To assess the impact on pericyte contractility—a key determinant of microvascular tone—single‐cell responses to carbachol stimulation were analyzed by time‐lapse microscopy. Under normoxic conditions, all groups displayed comparable contraction, characterized by protrusion retraction and intercellular gap formation (Figure S2f‐g). Under hypoxic conditions, however, cNGR‐EVs treatment preserved HBVP contractility compared to control EVs (Figure S2f‐g).

To further validate targeting in non‐transformed cells, uptake experiments were performed in primary human brain pericytes (PCs). Flow cytometry with WM15 antibody showed that nearly 100% of PCs were CD13‐positive (Figure S2h). As shown in Figure 2f, Cy3‐cNGR‐EVs exhibited enhanced uptake by PCs compared to Cy3‐EVs and Cy3‐Scr‐EVs, consistent with HBVP results. To determine whether O‐glycans affect cNGR recognition, PCs were treated with neuraminidase (NA) to remove terminal sialic acids and O‐glycans (Figure S2i). NA treatment did not affect Cy3‐cNGR‐EV association with PCs (Figure 2f). Functional analysis in PCs showed no differences in contractility under normoxia (Figure S2j). Under hypoxia, however, cNGR‐EVs enhanced contractile responses compared to control EVs (Figure 2g). These data indicate that cNGR functionalization promotes preferential association of EVs with CD13‐expressing pericytes and supports their contractile function under hypoxic stress.

### 3.3 CNGR Functionalization Preserves Intranasal EVs Biodistribution While Enhancing in Vivo Pericyte Targeting

After validating the CD13‐targeting capability and pericyte‐modulating effects of cNGR‐EVs *in vitro*, we next evaluated their biodistribution in mice following intranasal administration. For higher contrast and better signal‐to‐noise ratio, Cy7‐labeled EVs variants were tracked using NIRF imaging instead of Cy3‐labeled EV variants. Consistent with our previous findings on the temporal distribution patterns of intranasal EVs (Shen et al. 2023), Cy7 signals were detected in the brain 2 h post‐administration (Figure S3a‐b). In contrast, only minimal fluorescence was observed in peripheral organs, further confirming the efficient brain penetration of intranasally delivered EVs. Both Cy7‐cNGR‐EVs and Cy7‐Scr‐EVs exhibited similar organ distribution patterns (Figure S3a‐b). No statistically significant differences were observed in brain accumulation between Cy7‐cNGR‐EVs groups and Cy7‐EVs groups (Figure S3b). These results demonstrate that cNGR functionalization maintains the inherent biodistribution properties of intranasally administered EVs while not significantly altering their *in vivo* trafficking patterns.

**FIGURE 3:**
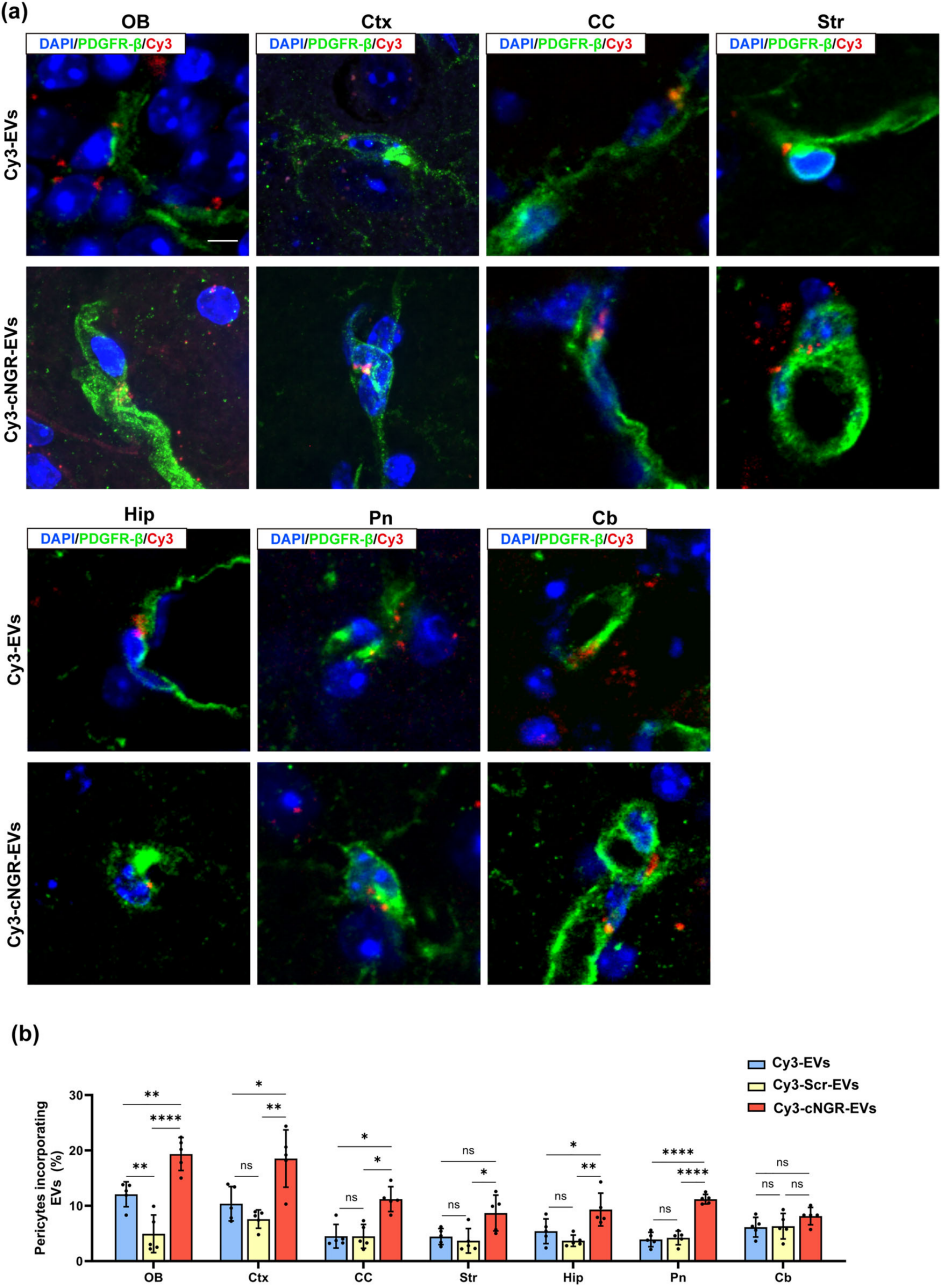
**Regional pericytes localization of intranasally administered Cy3‐cNGR‐EVs. (a)** Representative confocal microscopy images (high magnification) showing the uptake of Cy3‐EVs and Cy3‐cNGR‐EVs (red) by PDGFRβ^+^ pericytes (green) across different brain regions. Corresponding low‐magnification images showing the anatomical distribution of Cy3 fluorescence and the imaging data for Scr‐EVs uptake are provided in Figure S4 and Figure S5, respectively. Scale bar: 4 µm. **(b)** Quantitative analysis of EVs uptake in pericytes across brain regions. Data are represented as mean ± SD, *n* = 5 mice/group. Detailed statistical information is provided in the source data Table S2. Significance levels: *P < 0.05, **P < 0.01, ***P < 0.001, ****P < 0.0001. Abbreviations: OB, olfactory bulb; Ctx, cortex; CC, corpus callosum; Str, striatum; Hip, hippocampus; Pn, pons; Cb, cerebellum.

After characterizing the intranasal distribution patterns of EVs, we further investigated the *in vivo* pericyte‐targeting capability of cNGR‐EVs using immunofluorescence imaging. At 2 h post‐intranasal administration, Cy3^+^ punctuate signals were observed in multiple brain regions in all EVs‐treated groups (Cy3‐EVs, Cy3‐Scr‐EVs, and Cy3‐cNGR‐EVs), whereas no signals were detected in the control mice (Figure 3, Figure S4‐5). Following Cy3‐cNGR‐EVs administration, immunofluorescence imaging revealed increased association of Cy3^+^ punctuate signals with pericytes across various brain regions (Figure 3a). Quantitative analysis showed that Cy3‐cNGR‐EVs exhibited significantly higher pericyte association than Cy3‐EVs (Figure 3b). This enhanced uptake occurred not only in the olfactory bulbs and pons, key entry points of the nose‐to‐brain pathway but also in functionally critical regions including the cortex, corpus callosum (CC), and hippocampus (Figure 3b). In contrast, Cy3‐Scr‐EVs failed to enhance pericyte uptake relative to Cy3‐EVs. These results demonstrate that cNGR functionalization significantly improves pericyte‐targeting of EVs *in vivo*, consistent with our *in vitro* findings.

### 3.4 Intranasal cNGR‐EVs Alleviate Hypoperfusion‐Induced Cognitive and Myelin Damage Through Pericyte Preservation

Building on the observed pericyte‐targeting and contractility‐preserving effects of cNGR‐EVs under hypoxia, we next evaluated their therapeutic potential in a BCAS model of chronic cerebral hypoperfusion. Three‐month‐old wild‐type mice underwent sham or BCAS surgery and received PBS, control EVs, or cNGR‐EVs. Behavioral and pathological assessments were performed on 30 days post‐operation.

**FIGURE 4:**
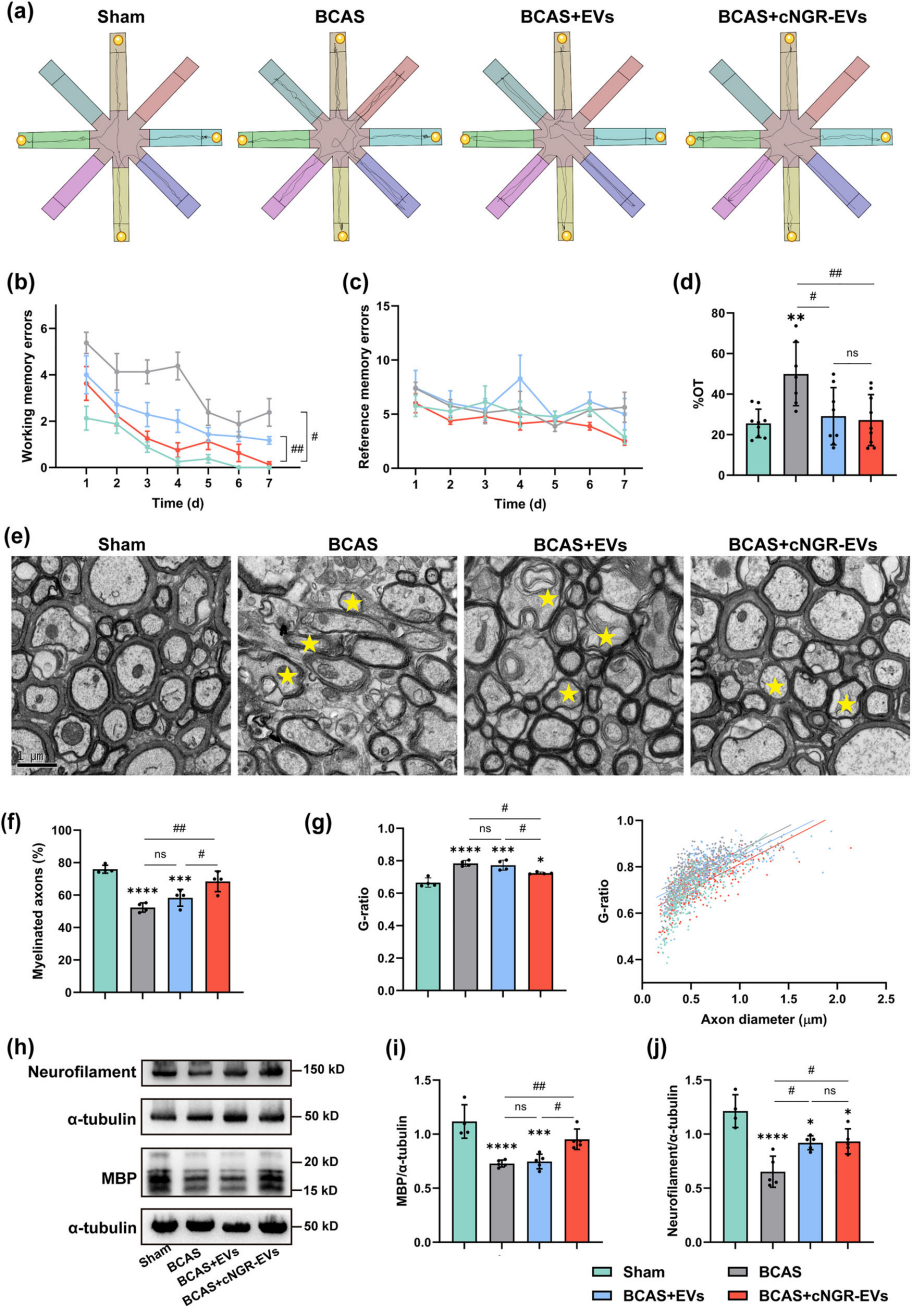
**Intranasal cNGR‐EVs attenuated cognitive deficits and demyelination in BCAS mice. (a)** Representative 8‐arm radial maze trajectories of Sham (sham‐operated), BCAS, BCAS + EVs (negative control EVs‐treated) and BCAS + cNGR‐EVs (cNGR‐EVs‐treated) mice on day 7 of testing. Yellow balls indicate baited arms. Mice in the BCAS group show impaired spatial memory (frequent revisits to any arm). **(b‐c)** Quantification of working memory errors (**b**) and reference memory errors (**c**) across groups: **(b)**Sham/BCAS/BCAS + cNGR‐EVs: *n* = 8 mice/group, BCAS + EVs: *n* = 7 mice. Mixed‐effects model with Tukey's test. Time: *P* < 0.0001, Intervention: *P* < 0.0001, Time×Intervention: *P* = 0.1594. On 7 days post testing, BCAS versus BCAS + EVs: *P* = 0.2799, BCAS versus BCAS + cNGR‐EVs: *P* = 0.027, BCAS + cNGR‐EVs versus BCAS + EVs: *P =* 0.0026. **(c)** Sham/BCAS/BCAS + cNGR‐EVs: *n* = 8 mice/group, BCAS + EVs: *n* = 7 mice. Mixed‐effects model Time: *P* = 0.0081, Intervention: *P* = 0.0628, Time × Intervention: *P* = 0.7024. **(d)** Quantitation of the percentage of open arm time (%OT) in the elevated plus maze (EPM). Sham/BCAS + cNGR‐EVs: *n* = 9 mice, BCAS: *n* = 7 mice, BCAS + EVs: *n* = 8 mice. One‐way ANOVA with Tukey's test. **(e)** Representative TEM images of corpus callosum (CC) at 30 days post‐surgery. Yellow stars indicate demyelinated axons exhibiting loosely wrapped lamellae or interlamellar cavities. Scale bar: 1 µm. **(f)** Quantification of the percentage of myelinated axons. *n* = 4 mice/group. **(g)** Myelin thickness analysis *via* g‐ratio (left) across groups. *n* = 4 mice/group. G‐ratio relationship to axon diameter (right) with lines representing linear regressions of each group and individual points correspond to single myelinated axons. Sham:Y = 0.3275 × X + 0.5238, BCAS: Y = 0.2099 × X + 0.6611, BCAS + EVs: Y = 0.2062 × X + 0.6380, BCAS + cNGR‐EVs:Y = 0.2159 × X + 0.5961. **(h)** Immunoblot of myelin basic protein (MBP), neurofilament and α‐tubulin expression in CC lysates across groups. **(i‐j)** Quantification of MBP **(i)** and neurofilament **(j)** protein levels. Sham: *n* = 4 mice, BCAS/BCAS + EVs/BCAS+cNGR‐EVs: *n* = 5 mice/group. One‐way ANOVA with Tukey's test. Data are presented as mean ± SD. Detailed statistical information is provided in the source data Table S3. Significance levels: *P < 0.05, **P < 0.01, ***P < 0.001, ****P < 0.0001 versus Sham; #*P* < 0.05, ##*P* < 0.01, ###*P* < 0.001, ####*P* < 0.0001.

In the 8‐arm radial maze, BCAS mice exhibited pronounced working memory deficits, repeated entries into any arm more frequently than sham controls (Figure 4a). While EVs treatment partially alleviated these deficits, cNGR‐EVs showed a superior effect in mitigating working memory impairment compared to both PBS and EVs treated groups (Figure 4b). The BCAS group did not exhibit a significant deficit in reference memory compared to the sham group, consistent with the literature (Shibata et al. 2007) (Figure 4c). Anxiety‐like behaviors were assessed using the elevated plus maze (Figure S6a‐d), revealing no intergroup differences in locomotor activity (Figure S6b‐d). Despite this, BCAS mice exhibited significantly increased open arm exploration time, indicative of abnormal risk‐taking (Figure 4d). Treatment with either EVs or cNGR‐EVs preserved this behavior at sham‐like levels (Figure 4d), further supporting the protective potential of EVs.

Demyelination represents a hallmark pathological feature of BCAS. Consistent with this, TEM analysis of the CC 30 days post‐surgery revealed more aberrant myelin structures (Figure 4e) and a marked reduction in myelinated axon density in BCAS‐operated mice compared to sham controls (Figure 4f). cNGR‐EVs provided robust protection, markedly preserving myelinated axon density, whereas control EVs failed to confer such an effect (Figure 4f). Demyelinated fibers in BCAS mice displayed elevated g‐ratios (myelin sheath thickness) and an upward shift in the g‐ratio/axon diameter distribution. These pathological alterations were significantly attenuated by cNGR‐EVs treatment, which maintained g‐ratios at levels closer to those of sham controls (Figure 4g). Western blot further confirmed substantial downregulation of MBP and neurofilament in BCAS mice, indicative of demyelination and concomitant axonal injury (Figure 4h‐j). Notably, cNGR‐EVs significantly maintained MBP levels, whereas control EVs did not (Figure 4i). Both EVs and cNGR‐EVs partially preserved neurofilament levels, suggesting the maintenance of axonal integrity (Figure 4j).

**FIGURE 5:**
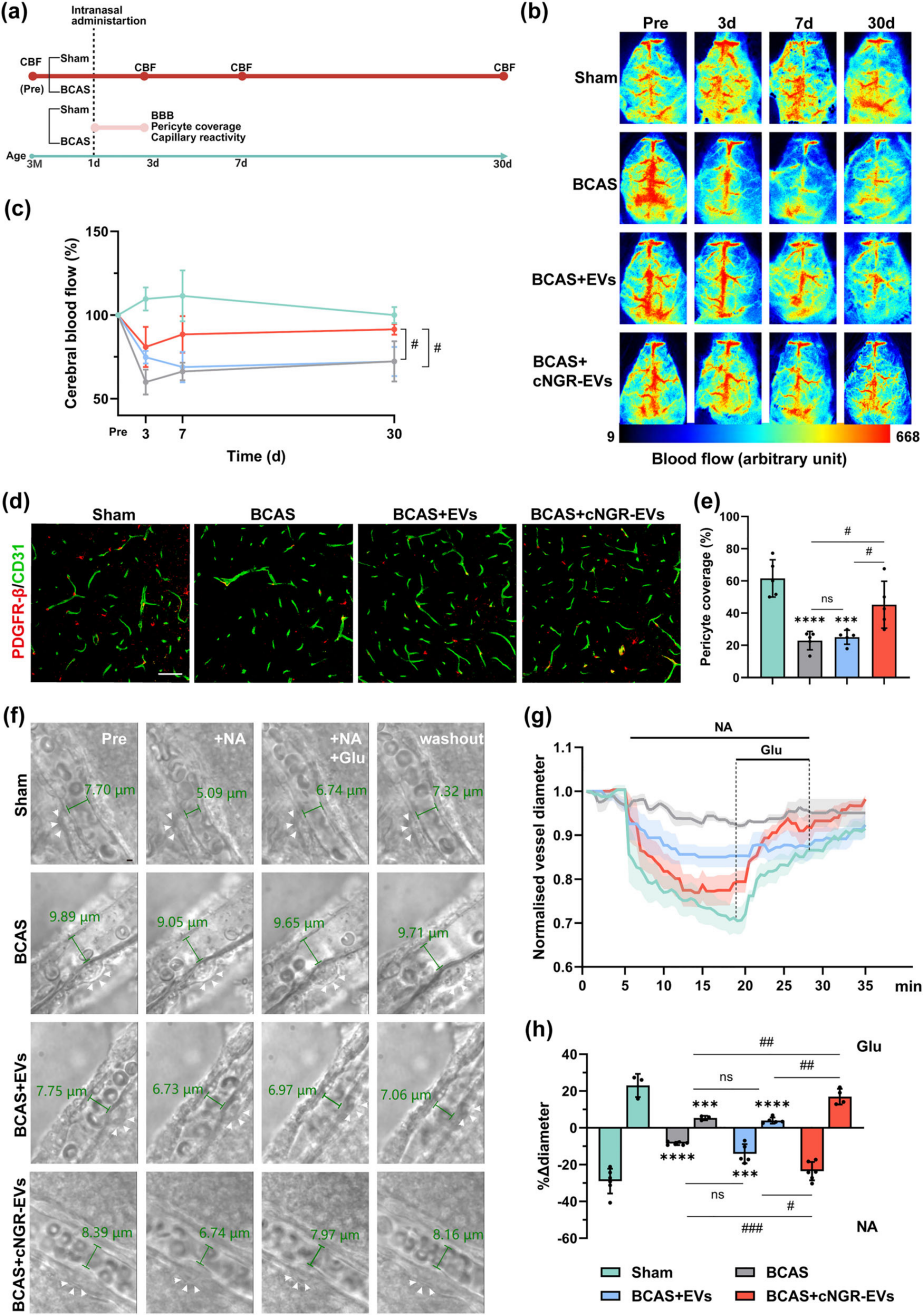
**Intranasal cNGR‐EVs preserved CBF and capillary reactivity in BCAS mice. (a)** Experimental timeline of study design. Three‐month‐old mice were subjected to BCAS surgery. Intranasal administration of either EVs or cNGR‐EVs was initiated on the day of surgery. CBF was measured on post‐operative day 3, 7, and 30. Additional analyses, including BBB permeability, pericyte coverage and vasoreactivity, were assessed on day 3. **(b)** Representative laser speckle contrast images of CBF at baseline and 3, 7, and 30 days post‐operation. **(c)** Quantification of CBF over time (% of preoperative baseline) across groups. Sham/BCAS/BCAS + EVs: *n* = 6 mice/group, BCAS + cNGR‐EVs: *n* = 8 mice. Mixed‐effects model with Tukey's test. Time: *P* < 0.0001, Intervention: *P* < 0.0001, Time × Intervention: *P* < 0.0001. On 30 days post‐operation, BCAS versus BCAS + EVs: *P* > 0.9999, BCAS versus BCAS + cNGR‐EVs: *P* = 0.0381, BCAS + cNGR‐EVs versus BCAS + EVs: *P =* 0.0208. **(d)** Immunofluorescence of pericyte (PDGFRβ^+^) and endothelial cells (CD31^+^) colocalization (pericyte coverage) in brain microvasculature. Scale bar: 50 µm. **(e)** Quantification of pericyte coverage. *n* = 5 mice/group, One‐way ANOVA with Tukey's test. **(f)** Live imaging of capillary dynamics in acute brain slices. White triangles indicate pericyte somata. Sequential conditions: baseline (Pre), superfusion with 2 µM noradrenaline (+NA), co‐superfusion with 2 µM NA + 500 µM glutamate (+NA+Glu), and washout. Green lines indicate the capillary lumen used for diameter measurements. Scale bar: 2 µm. **(g)** Time‐course of capillary diameter changes (shaded areas). Responses normalized to baseline diameter. **(h)** Quantification of peak capillary diameter changes, including glutamate‐evoked dilation (upper) and noradrenaline‐evoked constriction (lower). NA condition: Sham/BCAS/BCAS + cNGR‐EVs: *n* = 6 mice/group, BCAS+EVs: *n* = 5 mice; Glu condition: Sham/BCAS: *n* = 3 mice/group, BCAS + EVs: *n* = ‐5 mice, BCAS + cNGR‐EVs: *n* = ‐4 mice; One‐way ANOVA with Tukey's test. Data are presented as mean ± SD. Detailed statistical information is provided in the source data Table S4. Significance levels: ***P < 0.001, ****P < 0.0001 versus Sham; #*P* < 0.05, ##*P* < 0.01, ###*P* < 0.001.

To further elucidate the mechanisms by which cNGR‐EVs ameliorate behavioral deficits and demyelination in BCAS mice, CBF was monitored longitudinally at 3, 7, and 30 days post‐operation. Consistent with previous reports of early BBB disruption and pericyte loss (Liu et al. 2019), BBB permeability, pericyte coverage, and vascular reactivity were further assessed at day 3 (Figure 5a). Laser speckle flowmetry confirmed a marked reduction in CBF in BCAS mice, which declined to 59.9% of baseline levels by day 3 (Figure 5b‐c). Both EVs and cNGR‐EVs significantly mitigated this decline, maintaining CBF at ∼74.8% and ∼81.0% of baseline, respectively. While neither treatment affected CBF in sham‐operated mice (Figure S7a), suggesting that their effects are specific to the hypoperfused condition. However, the protective effect of control EVs was transient, with CBF dropping to levels comparable to BCAS controls by day 7. In contrast, cNGR‐EVs exerted a sustained stabilization of CBF, with levels remaining higher throughout the 30‐day observation period, compared to EVs‐treated mice (Figure 5c). On day 3, BCAS induced marked BBB leakage aned pericyte loss in both cortical and subcortical regions (Figure S7b‐e, Figure 5d‐e). Both EVs and cNGR‐EVs reduced BBB disruption across different brain regions, including CC (Figure S7b‐e), whereas preservation of pericyte coverage was more evident in the cNGR‐EVs‐treated group (Figure 5d‐e). These findings suggest that cNGR‐EVs may maintain BBB integrity with pericyte protection.

To further validate this hypothesis, we examined whether cNGR‐EVs directly modulate pericyte function *in vivo* by assessing pericyte‐mediated vascular responses using living brain slice imaging. In sham mice, capillaries demonstrated robust constriction in response to noradrenaline (Figure 5f‐h), followed by glutamate‐evoked dilation superimposed on baseline vascular tone, with maximal responses localized near pericyte somata (Figure 5f). BCAS brain slices showed markedly attenuated constriction and dilation kinetics with smaller diameter changes compared to the sham group, indicating impaired vascular reactivity (Figure 5g). Control EVs partially preserved contractile responses but failed to maintain glutamate‐induced dilation (Figure 5g). Conversely, cNGR‐EVs preserved near‐normal sequential constriction and dilation in response to noradrenaline and glutamate challenge (Figure 5g), with response amplitudes significantly greater than those in both BCAS and BCAS + EVs groups (Figure 5h). Collectively, these findings indicate that cNGR‐EVs confer multimodal protection on CBF, BBB integrity, and capillary reactivity in the BCAS model, in association with preservation of pericyte function.

### 3.5 CNGR‐EVs Partially Ameliorate Pericyte‐Associated Transcriptomic Alterations and Maintain Neurovascular Communication After Chronic Hypoperfusion

**FIGURE 6:**
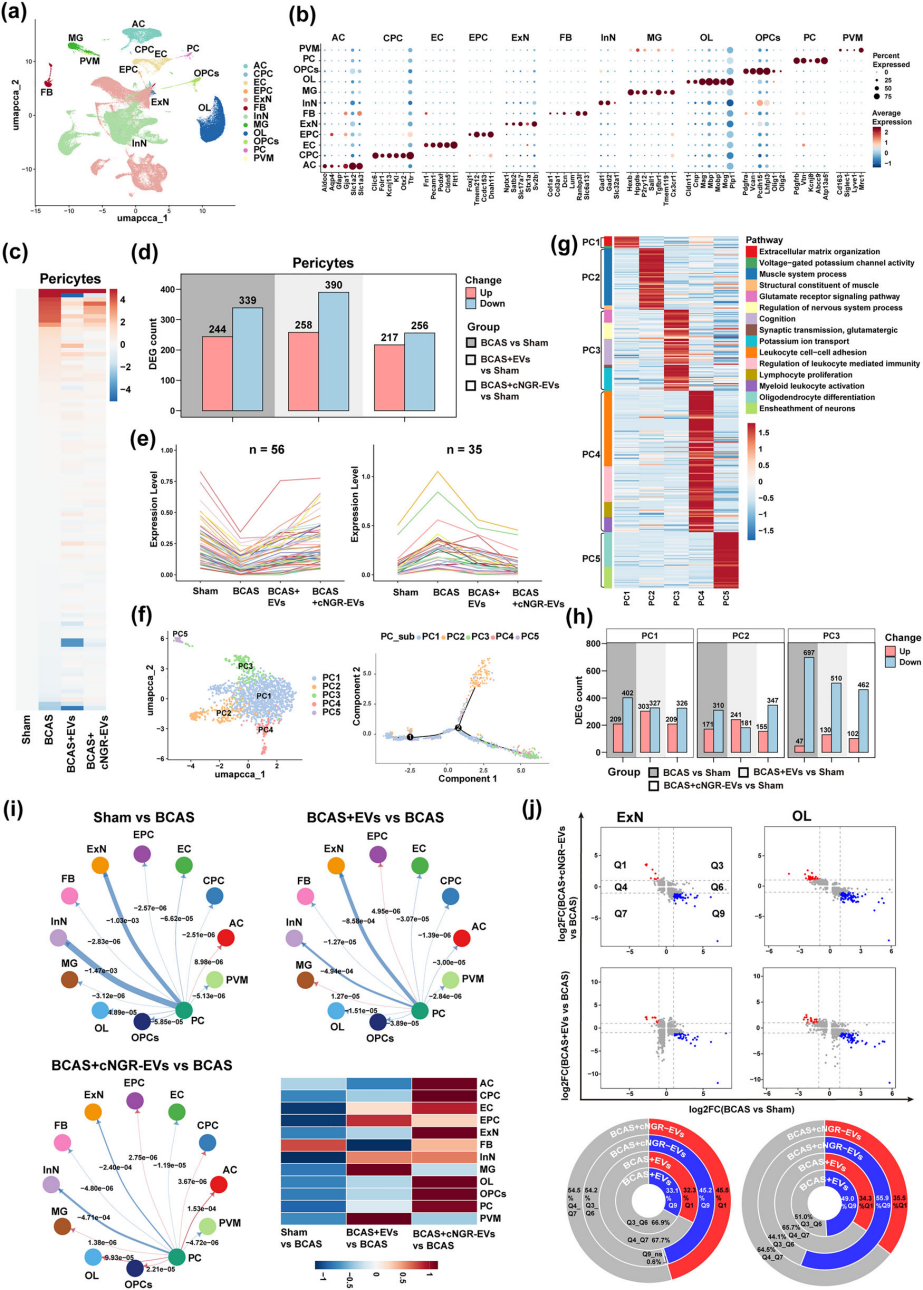
**cNGR‐EVs therapy blunted pericyte‐associated transcriptomic alterations induced by BCAS. (a)** UMAP visualization of single‐cell RNA‐seq data, with cells colored by different types. **(b)** Proportion and average expression levels of canonical marker genes across identified cell types. **(c)** Heatmap of log_2_‐transformed relative normalized expression of pericytes differentially expressed genes (DEGs) across Sham, BCAS, BCAS + EVs, and BCAS + cNGR‐EVs groups, normalized to the Sham control. **(d)** Number of DEGs in pericytes identified in BCAS versus Sham, BCAS + EVs versus Sham, and BCAS + cNGR‐EVs versus Sham comparisons. **(e)** Line plots illustrating the expression patterns of DEGs in pericytes across groups Left: 56 genes downregulated in BCAS and upregulated following cNGR‐EV treatment. Right: 35 genes upregulated in BCAS and reduced following cNGR‐EV treatment. *N* represents the number of genes in each cluster. **(f)** UMAP visualization (left) identifies five distinct pericyte sub‐clusters (PC1–PC5). Pseudotime trajectory analysis (right) showing the progression of pericyte states, rooted at node 1 (initial stage), progressing through node 2 (intermediate stage), and eventually diversifying into terminal branches (late stages). **(g)** Gene Ontology (GO) enrichment analysis of upregulated genes in pericyte subclusters. **(h)** Number of DEGs in pericyte subclusters (PC1–PC3) across BCAS versus Sham, BCAS + EVs versus Sham, and BCAS+cNGR‐EVs versus Sham comparisons. **(i)** Cell–cell communication analysis showing changes in interaction strength between pericytes and other brain cell types. The maps represent the subtraction of interaction strengths: Sham vs BCAS, BCAS + EVs versus BCAS, and BCAS + cNGR‐EVs versus BCAS. Blue and red lines indicate decreased or increased interaction strength, respectively. A heatmap summarizes overall interaction changes. **(j)** Nine‐quadrant plots (up) comparing transcriptomic changes (log2 Fold Change) induced by BCAS (*x*‐axis) and treatment (*y*‐axis; BCAS + cNGR‐EVs or BCAS + EVs) in excitatory neurons and oligodendrocytes. Genes in Q1 (downregulated by BCAS but upregulated by treatment) and Q9 (upregulated by BCAS but downregulated by treatment) represent significantly reversed DEGs (highlighted in red and blue, respectively). Nested pie charts (bottom) illustrating the percentage of genes in each quadrant. The outer two rings represent the cNGR‐EVs group, while the inner two rings represent the EVs group. The larger areas of Q1 (red) and Q9 (blue) in the outer rings indicate that cNGR‐EVs achieved a more comprehensive preservation of the transcriptomic profile compared to EVs in both excitatory neurons and oligodendrocytes. Abbreviations: ExN, excitatory neurons; InN, inhibitory neurons; OL, oligodendrocytes; AC, astrocytes; MG, microglia; OPCs, oligodendrocyte progenitor cells; EC, endothelial cells; PC, pericytes; CPC, choroid plexus cells; EPC, ependymocytes; FB, fibroblasts; PVM, perivascular macrophages.

To investigate the molecular mechanisms underlying cNGR‐EVs‐mediated pericyte regulation, we performed single‐nucleus RNA sequencing (snRNA‐seq) on whole brains samples from BCAS mice treated intranasally with PBS, control EVs, cNGR‐EVs, with sham‐operated littermates as controls. Unsupervised clustering identified 55 clusters corresponding to 12 major cell types, including excitatory neurons, inhibitory neurons, oligodendrocytes, astrocytes, microglia, oligodendrocyte progenitor cells (OPCs), endothelial cells, and pericytes etc., based on canonical marker genes (Figure 6a‐b, Figure S8a). All major cell populations were detected across groups (Figure S8b‐e). Compared with Sham controls, BCAS mice showed changes in cell‐type composition, including an increased proportion of excitatory neurons and a modest increase in pericyte representation, which appeared partially normalized following EV and cNGR‐EV treatment (Figure S8d‐e). Differential expression analysis revealed widespread transcriptional alterations across cell types in BCAS mice relative to sham controls (Figure 6c–d, Figure S8f). In pericytes, cNGR‐EVs were associated with a greater reduction in the number of differentially expressed genes (DEGs) compared to control EVs, and gene expression patterns showed a trend towards normalization (Figure 6c–e).

To resolve pericyte transcriptional heterogeneity, subclustering analysis identified five pericyte subpopulations (PC1–PC5) with distinct transcriptional profiles (Figure 6f, Figure S9a). BCAS was associated with shifts in subpopulation composition, including reduced PC1 fractions and increased PC3 abundance, whereas cNGR‐EV treatment showed a distribution pattern similar to that of the sham group (Figure S9b). Pseudotime analysis suggested a continuum of pericyte states across conditions (Figure 6f, Figure S9c). Gene ontology (GO) and KEGG pathway enrichment analyses revealed distinct functional signatures across pericyte subclusters (Figure 6g, Figure S9d). For example, PC1 (the dynamic cluster) was enriched for genes related to extracellular matrix organization, potassium channel activity and neurodegeneration. PC2‐PC5 enrichment for gene programs associated with contraction, neurovascular signaling, immune‐related processes, or glial interaction.

We further analyzed the number of DEGs relative to the Sham group across all pericyte sub‐clusters (Figure 6h, Figure S9e). cNGR‐EVs treatment was associated with a reduction in DEG burden relative to BCAS and EV‐treated groups. This restoration of the DEG pool suggests that cNGR‐EVs stabilized the transcriptomic profile of pericytes, maintaining them in a state closer to physiological homeostasis following BCAS‐induced injury. Notably, Ttr expression displayed consistent changes across conditions in both total pericytes and the PC1 subcluster (Figure S9f), suggesting a potential association with pericyte state transitions. Enrichment analyses of reversal genes in cNGR‐EVs‐treated groups preserved distinct biological processes across subpopulations (Figure S9g).

To assess changes in intercellular communication of NVU, we performed differential cell‐cell communication analysis (Figure 6i). BCAS was associated with widespread alterations in predicted interaction strength between pericytes and other cell types. cNGR‐EV treatment partially ameliorated these interaction patterns, particularly in pathways involving neurons, oligodendrocytes, and astrocytes, whereas control EVs showed more limited effects. This protective effect was further confirmed by the heatmap of overall interaction strengths. Nine‐quadrant analysis further indicated that cNGR‐EVs were associated with a higher proportion of genes exhibiting opposite‐direction changes relative to BCAS in neurons, oligodendrocytes, OPCs and astrocytes (Figure 6j, Figure S10). Specifically, a substantial proportion of DEGs were located in quadrants Q1 and Q9, representing genes whose expression was preserved by the treatment. Nested pie charts further quantified this effect: the outer rings (cNGR‐EVs) exhibited a higher percentage of significantly reversed genes (Q1 and Q9) compared to the inner rings (EVs), demonstrating that cNGR modification significantly enhances the protective potential of EVs across multiple cell populations.

## 4 Discussion and Conclusion

Despite the high prevalence of VCI, disease‐modifying therapies remain lacking. Current treatment is largely extrapolated from Alzheimer's disease and primarily includes cholinesterase inhibitors and NMDA receptor antagonists, which provide modest symptomatic benefit but do not address the underlying vascular pathology (Ip et al. 2024). Management also focuses on controlling vascular risk factors and preventing cerebrovascular events, but these approaches have limited efficacy in halting disease progression. Emerging evidence suggests that vascular cell dysfunction plays a critical role in VCI pathogenesis, with pericytes being key regulators of CBF and BBB integrity (Ip et al. 2024). In this context, our pericyte‐targeted EV strategy provides a mechanistically focused approach to protect vascular function and mitigate downstream neurodegenerative changes. In this study, we present a bio‐orthogonal chemistry‐mediated approach for the conjugation of EVs with cNGR peptides, enabling the development of a novel pericyte‐targeted therapy for VCI characterized by early‐stage pericyte dysfunction.

Chemical modification is a widely used strategy for engineering the EV surface (Li et al. 2025). Unlike genetic engineering of parent cells, it offers advantages by enabling direct modification of pre‐isolated EVs from various sources, and avoiding the potential risks of introducing foreign genetic material (Richter et al. 2021). The critical challenge of this approach, however, lies in creating stable linkages between targeting ligands and the EV surface without compromising vesicle integrity or the bioactivity of membrane proteins (Richter et al. 2021). Among these, SPAAC is widely used due to its copper‐free nature, thereby avoiding potential oxidative damage associated with Cu(I)‐catalyzed reactions (Richter et al. 2021). In the present study, cNGR peptides were conjugated to EVs *via* a bio‐orthogonal SPAAC reaction between DBCO‐modified EV membranes and azide‐functionalized ligands. Although EVs modified *via* SPAAC undergo a modest increase in size and exhibit some heterogeneity in biomechanical properties, the method has been consistently validated as biocompatible and non‐toxic, preserving fundamental EV functions. Furthermore, SPAAC‐based modification offers a more straightforward procedure while generating stronger and more stable covalent bonds than non‐covalent interactions (Attia et al. 2024; Pham et al. 2021). This resultant stability guarantees prolonged circulation of half‐life of functionalized EVs, thereby improving their delivery efficiency to target cells.

Achieving cell‐type specificity remains a major challenge in EV‐based delivery. Most currently available brain‐targeting peptides are derived from neurotropic viruses or anti‐tumor ligands (Tang et al. 2025). For example, RVG29, a peptide originating from rabies virus glycoprotein, binds nicotinic acetylcholine receptors on neurons and facilitates neuronal targeting (Liu et al. 2009). Similarly, RGD peptides (Arg‐Gly‐Asp) recognize αvβ3 and αvβ5 integrins, which are highly expressed on glioblastoma vasculature, thereby promoting endothelial uptake (Tian et al. 2018). However, rationally designed peptides with selective affinity for other CNS cell types remain exceedingly scarce, underscoring the need for innovative strategies to enhance the precision of EV targeting within the brain.

In this study, we repurpose NGR peptides for pericyte targeting, extending their established use in tumor therapy. Molecular docking suggested a favorable interaction between cNGR and CD13; however, it should be noted that available CD13 structures lack physiologically relevant glycosylation, which may influence ligand binding in vivo. With Scr‐EVs serving as a size‐matched non‐targeting control, cNGR‐EVs demonstrated markedly increased uptake by brain pericytes both in vitro and in vivo, supporting preferential targeting, although minor off‐target uptake by other CD13^+^ cell types cannot be excluded. Future studies should quantify the cellular distribution of cNGR‐EVs in the brain.

While intravenous administration remains common, it poses notable limitations for CD13‐targeted delivery due to off‐target binding in peripheral organs—particularly the lungs—and the formation of plasma protein coronas that can obscure targeting peptides and accelerate clearance (Tóth et al. 2021; Wiklander et al. 2015). In contrast, intranasal delivery offers a direct route to the brain, largely bypassing the BBB and minimizing systemic exposure (Lochhead and Thorne 2012). Consistent with our previous findings, intranasally administered EVs rapidly reached the brain within 2 h and showed predominant cerebral accumulation (Shen et al. 2023). This route enabled efficient transport along cerebrospinal fluid pathways and facilitated perivascular pericyte uptake (Shen et al. 2023). Quantitative imaging further demonstrated significantly enhanced brain retention and pericyte uptake of cNGR‐EVs compared with control EVs, confirming the effectiveness of this in vivo targeting strategy. It should be noted that hyaluronidase pretreatment, used to facilitate intranasal delivery, may introduce confounding factors such as local mucosal effects or altered absorption kinetics. Although the therapeutic efficacy of cNGR‐EVs was evaluated in BCAS mice following a single intranasal administration immediately after surgery, potential alterations in vascular architecture under hypoxic conditions may influence targeting efficiency and cannot be excluded. In addition, while biodistribution was assessed in healthy animals, and potential differences under pathological conditions, such as hypoperfusion, warrant further investigation.

Previous studies have established that pericytes play a critical role in maintaining capillary basal tone (Østergaard et al. 2015) and their dysfunction disrupts local microvascular tension, leading to heterogeneous blood flow and impaired oxygen extraction (Hartmann et al. 2022). In the BCAS model, loss of vascular tone and reduced arteriolar contractility have been linked to pericyte dysfunction, contributing to chronic hypoperfusion–induced demyelination (Kim et al. 2021). Consistently, in this study, we observed a marked reduction in CBF following BCAS, accompanied by decreased pericyte coverage and impaired ischemia‐evoked capillary contractility. Intranasal administration of cNGR‐EVs effectively attenuated the acute postoperative CBF decline and maintained long‐term hemodynamic stability, preserving pericyte coverage and their contractile responsiveness. Ex vivo live brain slice experiments further showed that capillaries from cNGR‐EVs‐treated mice exhibited faster and more robust contractile and dilatory responses to noradrenaline and glutamate at pericyte sites. Importantly, cNGR‐EVs did not alter CBF in sham‐operated mice, indicating that their effects are context‐dependent and more prominent under hypoperfused conditions. Together, these findings support the notion that cNGR‐EVs safeguard microvascular function, likely through modulation of pericyte activity.

It is worth noting that in the present study, cNGR‐EVs were administered concurrently with the induction of chronic hypoperfusion. This experimental design demonstrates the promising neuroprotective and preventative capacity of cNGR‐EVs in mitigating the onset of VCI pathology. As noted by the early preservation of CBF at day 3, the intervention likely functions by blunting the initial cascade of microvascular damage. However, they do not yet constitute evidence of repairing pre‐existing vascular demyelination. Future investigations should evaluate the therapeutic efficacy of cNGR‐EVs when administered at more advanced stages of pathology to further explore their potential for restorative intervention.

In addition to CBF regulation, both control EVs and cNGR‐EVs attenuated BBB disruption following BCAS; however, only cNGR‐EVs preserved pericyte coverage compared to BCAS and EV‐treated groups. This differential effect suggests that targeted modulation of pericytes may contribute to BBB stabilization. Notably, the regional differences observed between Evans Blue and albumin extravasation assays likely reflect methodological variations. Evans Blue quantification is more susceptible to residual intravascular dye despite perfusion, whereas albumin immunostaining directly visualizes parenchymal leakage and may provide a more region‐specific assessment. Such discrepancies are not unexpected and are consistent with the spatial heterogeneity of BBB impairment in chronic cerebral hypoperfusion.

Unbiased single‐cell transcriptomics further support the mechanistic role of cNGR‐EVs in pericyte modulation. Notably, the transcriptomic changes induced by cNGR–‐EVs was not uniform across all pericytes but varied across distinct sub‐cluster, suggesting cell state–dependent responses. Among the genes affected, Ttr—a thyroid hormone transporter primarily synthesized in liver and choroid plexus—showed consistent downregulation following cNGR‐EV treatment across total pericytes and the major subcluster (Liz et al. 2020). Although pericyte expression of Ttr is rarely reported, emerging evidence indicates its involvement in neuroprotection, including neurite outgrowth, Aβ clearance, and remyelination through thyroid hormone supply (Corino et al. 2025; Pagnin et al. 2022; Yang et al. 2022). In contrast, other studies suggest that Ttr acts as a negative regulator of OPC proliferation and remyelination, indicating that its regulation appears context‐dependent (Alshehri et al. 2020; Pagnin et al. 2022). In this study, changes in Ttr expression may reflect alterations in pericyte functional states; however, its precise role in pericytes remains to be determined. Importantly, we cannot distinguish whether the observed normalization of neuronal and glial transcriptomes arises from direct pericyte‐mediated signaling or indirect effects secondary to improved CBF and BBB integrity. These mechanisms are likely interconnected and warrant further investigation.

Despite the protective effects of cNGR‐EVs observed in the BCAS model, several limitations should be acknowledged. First, dose/time–response relationships for EV uptake and functional effects were not systematically evaluated. Optimization of dosing strategies and comprehensive pharmacokinetic characterization will be necessary for future translational development. Second, although cNGR functionalization enhanced pericyte targeting, it also modified EVs surface charge and biomechanical properties, potentially affecting colloidal stability, biodistribution, and pharmacokinetics—parameters that remain incompletely characterized in vivo. Third, the specific EV cargo responsible for the observed effects was not identified, and future studies integrating proteomic and transcriptomic profiling will be necessary to define key mediators. Fourth, while snRNA‐seq revealed broad transcriptomic restoration and reprogramming of pericyte‐mediated intercellular networks, the precise molecular mechanisms underlying these effects, including the contributions of key effectors such as Ttr, require additional functional validation. Finally, the present study does not examine the effects of cNGR‐EVs treatment on OPCs differentiation or remyelination kinetics. Although improved myelin integrity was observed, the relative contributions of remyelination versus reduced demyelination were not directly assessed.

In summary, our study highlights pericytes as a critical therapeutic target in VCI and demonstrates that bio‐orthogonal cNGR functionalization enables preferential delivery of EVs to these cells. Intranasal administration of cNGR‐EVs improves microvascular function and is associated with downstream neuroprotective effects under chronic hypoperfusion. These findings support the feasibility of engineering EVs for cell‐type–directed delivery in the CNS and provide a potential framework for developing targeted therapies for vascular‐related neurodegenerative disorders.

## Author Contributions

**Weiwei Shen**: conceptualization, methodology, investigation, visualization, writing – original draft. **Weishi Liu**: methodology, investigation, visualization, writing – review and editing. **Min Guo**: methodology, visualization, funding acquisition, writing – review and editing. **Tongyao You**: methodology, investigation. **Yingzhe Wang**: methodology, funding acquisition. **Tiansiyu Wen**: investigation. **Suzhen Liang**: investigation. **Xiaodi Xie**: investigation. **Yanfeng Jiang**: methodology, funding acquisition. **Qiang Dong**: conceptualization, project administration, supervision, writing – review and editing. **Jin‐Tai Yu**: conceptualization, project administration, supervision, writing – review and editing. **Mei Cui**: conceptualization, supervision, project administration, writing – review and editing, funding acquisition.

## Funding

Ministry of Science and Technology of China (STI2030‐Major Projects2030, 2021ZD0201806 to Mei Cui). Natural Science Foundation of China (82271221 to Mei Cui, 82101336 to Min Guo, 82373658 to Yanfeng Jiang). Shanghai Municipal Health Commission (20234Z0013 to Mei Cui). Shanghai Oriental Talent Program (to Mei Cui). Shanghai Medical New Star Program (to Mei Cui, Min Guo, Yingzhe Wang). Shanghai Municipal Health Commission Clinical Research Special Program for the Health Industry (20244Y0103 to Yingzhe Wang, 20254Y0004 to Min Guo).

## Conflicts of Interest

The authors declareno conflict of interest.

## Supporting information




**Supplementary Methods**: Quantification workflows and sample size

**Supplementary Figures S1‐S10**: jev270319‐sup‐0002‐SuppMat.docx
Supplemented materials and methods

**Supporting Information**: jev270319‐sup‐0003‐TableS1.xlsx

**Supporting Information**: jev270319‐sup‐0004‐TableS2.xlsx

**Supporting Information**: jev270319‐sup‐0005‐TableS3.xlsx

**Supporting Information**: jev270319‐sup‐0006‐TableS4.xlsx

**Supporting Information**: jev270319‐sup‐0007‐TableS5.xlsx

**Supporting Information**: jev270319‐sup‐0008‐TableS6.xlsx

**Supporting Information**: jev270319‐sup‐0009‐TableS7.xlsx

**Supporting Information**: jev270319‐sup‐0010‐TableS8.xlsx

**Supporting Information**: jev270319‐sup‐0011‐TableS9.xlsx

**Supporting Information**: jev270319‐sup‐0012‐TableS10.xlsx

## Data Availability

All data are available in the main text or the supplementary materials. The raw sequence data reported in this paper have been deposited in the Genome Sequence Archive in China National Center for Bioinformation (GSA: CRA031526) that are publicly accessible at https://ngdc.cncb.ac.cn/gsa
