# Supplementary material for: Engineered Pericyte‐Targeted Extracellular Vesicles Protect Against Hypoperfusion‐Induced Cognitive Impairment and Vascular Demyelination
Source: J Extracell Vesicles. 2026 Jun 1;15(6):e70319. doi: 10.1002/jev2.70319 (PMC13240518; doi:10.1002/jev2.70319)
Supplement: Supplementary file 1 — Supplementary Methods: Quantification workflows and sample size [file JEV2-15-e70319-s008.docx]

**Quantification Workflow and Sample Size**

**Quantification Workflow**

**Blinding:**To ensure objectivity, all image acquisition and quantification were performed by an investigator blinded to the experimental groups. Image files were randomized and renamed with numeric codes before analysis.

1. **Pericyte Uptake of EVs** **(ICC)**

ROI Definition and Image Processing (ImageJ, v1.8.0): For each 3D confocal stack, a maximum intensity projection was generated to encompass the full intracellular EV signal. Orthogonal views (XZ and YZ planes) were utilized during preliminary inspection to confirm the internalization of EVs within the pericyte cytoplasm. Pericyte boundaries were defined using the CellMask channel. To minimize bias, cells were either traced manually or segmented using the MorphoLibJ based on the membrane signal. Each cell was added to the ROI Manager as an individual analytical unit. The multi-channel images were split to isolate the EV-specific channel (Cy3). A Rolling Ball algorithm (radius = 50 pixels) was applied to the EV channel to remove non-specific background noise and ensure signal-to-noise consistency. A fixed global threshold was established based on the Control group to effectively distinguish specific fluorescent puncta from background. For 16-bit images, the intensity range was strictly maintained at [1236, 65535] across all experimental groups to ensure comparability. The pre-defined cell ROIs were applied to the thresholded EV channel. The mean Cy3 intensity for each individual cell was measured.

Statistical Unit: For each independent experiment, a minimum of 5 random fields of view (FOVs) were captured per group, with at least 10~15 individual pericytes analyzed across these FOVs. The mean fluorescence intensities of all cells across these FOVs were pooled and averaged to represent the mean value for that independent biological replicate. Final statistical comparisons were performed based on the number of independent biological trials.

1. **Pericyte Contraction Assay**

ROI Definition and Image Processing (ImageJ, v1.8.0): Time-series images were imported. A Z-drift compensation (ZDC) system was utilized during capture to eliminate potential artifacts caused by axial focal fluctuations. This guarantees that all diameter quantifications were derived from the same optical section over time. Considering the inherent technical limitations of differential interference contrast (DIC) imaging—such as uneven illumination, "shadow-cast" effects, and halo artifacts which compromise the accuracy of automated segmentation—the cell boundaries were manually delineated. For each pericyte, the entire cell body and all radiating processes were traced at baseline (T_0_) using the Freehand Selection Tool and added to the ROI Manager. To ensure data quality, cells exhibiting signs of apoptosis, detachment, or overlapping with neighbors were strictly excluded. The same cell was tracked through the time-series, and its surface area was re-traced and measured at each subsequent time point (T_t_). %Retraction= [Area (T_0_) - Area (T_t_)]/ Area (T_0_) ×100%. All cells, including non-responding ones, were included in the analysis to provide an unbiased representation of the entire population.

Statistical Unit: For each independent experiment, a minimum of 5 random FOVswere captured per group, with at least 10~15 individual pericytes analyzed across these FOVs. The % Retraction values of all cells within these FOVs were pooled and averaged to generate a single representative mean for that independent biological replicate. Final statistical comparisons were performed based on the number of independent biological trials.

1. **Ex Vivo Organ Near-Infrared Imaging**

ROI Definition and Image Processing: Fluorescence images of excised organs were analyzed using the VISQUE In Vivo Smart-LF system (Vieworks) equipped with CleVue software (v3.1.3.2054). The quantitative metric used was radiant efficiency (p/sec/cm^2^/sr/[uW/cm^2^]), which provides a standardized measure of fluorescence emission normalized to the excitation light intensity. For each organ, the entire anatomical boundary was manually delineated using the Freehand Selection Tool to create a primary ROI. To account for local instrumentation noise and environmental light, a background ROI of equivalent size was defined in a non-tissue (blank) area adjacent to the organ. The net radiant efficiency was calculated by subtracting the mean radiant efficiency of the background ROI from that of the organ ROI. The net radiant efficiency of the Control group was defined as the baseline.

Statistical Unit: Three consecutive images were captured for each organ from each mouse. The relative radiant efficiency values from these three technical replicates were averaged to generate a single representative value for that specific organ. Final statistical comparisons were performed based on the number of mice.

1. **Regional Pericyte EV Uptake in Brain Slices (ICC)**

ROI Definition and Image Processing (ImageJ, v1.8.0): Brain regions were first localized at low magnification using a 10×objective lens to navigate according to the Paxinos and Franklin mouse brain atlas. For each mouse, at least 3 non-consecutive sagittal sections (separated by 120μm) were analyzed. Within each region of interest (ROI), at least 3 random FOVs were captured using a confocal microscope with a 60×, 100× objective. 3D Z-stacks were acquired. Maximum intensity projections were used for quantification, while orthogonal views (XZ/YZ) were periodically inspected. A Rolling Ball algorithm (radius = 50 pixels) was applied to both the EV and pericyte channels to eliminate diffuse background noise. A fixed global threshold was established for the EV channel based on the negative control group. A pericyte was defined as “EV-positive (EV+)” if it contained at least 1 distinct Cy3-fluorescent puncta within its cellular boundary. In each FOV, the total number of pericytes (N_total_, identified by PDGFRβ+/DAPI+) and the number of EV-containing pericytes (N_EV+_) were manually counted by an investigator blinded to the experimental groups. % pericyte incorporating EVs= N_EV+_/ N_total_ ×100%.

Statistical Unit: The percentages calculated from all FOVs within a specific brain region per mouse were averaged. Final statistical comparisons were performed based on the number of mice.

1. **Myelin G-ratio Quantification**

ROI Definition and Image Processing (ImageJ, v1.8.0): Digital TEM micrographs were calibrated using the Set Scale function in ImageJ. The pixel-to-micrometer (μm) conversion factor was determined based on the scale bar metadata provided by the microscope’s acquisition software, ensuring all linear measurements were physically accurate. To ensure the accuracy of the G-ratio, tissue blocks were meticulously oriented during embedding to ensure that sectioning was performed strictly perpendicular to the longitudinal axis of the nerve fibers. This resulted in near-perfect circular cross-sections. For each selected fiber, the inner axonal diameter (d) and the outer fiber diameter (D, including myelin) were measured directly using the Straight Line tool in ImageJ. For slightly elliptical axons, the short-axis diameter was used to represent the true diameter. G-ratio=d/D.

Statistical Unit: The G-ratio values of all axons within these FOVs were pooled and averaged to generate a single representative mean for that individual. Final statistical comparisons were performed based on the number of mice.

1. **Laser Speckle Contrast Imaging for Blood Flow Assessment**

ROI Definition and Image Processing: Cerebral blood flow (CBF) was monitored using the RWD Laser Speckle Imaging System (RWD Life Science). Data acquisition and post-processing were performed using the system’s proprietary analysis software. ROIs were manually delineated over the brain using the software’s ROI tool. The software automatically calculated the blood flow values within the ROIs, expressed in perfusion units (PU). To minimize variability, all measurements were conducted under identical anesthesia depth and room temperature control. To account for inter-individual physiological variability, a baseline measurement was recorded for each mouse prior to the surgery. This baseline value (PU_baseline_) was defined as 100%. Blood flow values at subsequent time points (PU_t_)were normalized to the individual’s baseline. The Mean Perfusion Units (PU) from these two technical replicates were averaged to represent the stable blood flow value for that specific time point. %Relative CBF=PU_t_/PU_baseline_ ×100%.

Statistical Unit: Final statistical comparisons were based on the number of mice, with each data point representing the normalized mean of the repeated measurements for one individual.

1. **Pericyte Coverage (ICC)**

ROI Definition and Image Processing: Multi-channel images were split, and the pericyte marker (PDGFR-β) and vascular marker (CD31) were converted into pseudo-colored 8-bit images. To minimize subjective bias and intra-class variance between black and white pixels, the Otsu’s auto-thresholding method was applied to channels. This created binary masks representing the specific fluorescent signals.The Raw Integrated Signal Density for each thresholded image was measured. To convert this density into a pixel-based area, the total integrated density was divided by 255 (the maximum pixel intensity for 8-bit images). The ratio of the pericyte-positive area (Area_PDGFR-β+_) over the endothelial-positive area (Area_CD31+_) was calculated to represent the percentage of pericyte coverage. % pericyte coverage= Area_PDGFR-β+_/Area_CD31+_

Statistical Unit: For each mouse, the coverage percentages from at least 5 random FOVs across multiple brain sections were pooled and averaged. Final statistical comparison was performed based on the number of mice.

1. **Quantification of Capillary Diameter Changes**

ROI Definition and Image Acquisition: Live imaging of acute brain slices was performed using an Olympus confocal system. Real-time vessel diameter changes were tracked using Olympus Oly VIA software (v2.9.1) and subsequently analyzed in ImageJ (v1.53). Capillaries (diameter < 10 μm) with clearly visible pericyte cell bodies (identified by DIC) were selected for analysis. To ensure consistency, the luminal diameter was measured at two specific positions immediately adjacent to both sides of the pericyte cell body. The average of these two measurements was recorded as the diameter for that single pericyte-vessel unit. Diameter measurements were recorded at three distinct stable phases: Baseline (d_Pre_): before any drug administration; Constriction (d_+NA_): at the peak response after Noradrenaline (NA) perfusion; Dilation (d_+NA+Glu_): at the peak response after Glutamate (Glu) perfusion following the NA-induced pre-constriction. The magnitude of vasomotor responses was quantified using the following standardized formulas:

NA-induced Constriction: NA %Δdiameter =(d_Pre_ - d_+NA_)/d_Pre_×100%

Glutamate-induced Dilation: Glu %Δdiameter= (d_+NA+Glu_ - d_+NA_)/d_Pre_×100%.

The Glu-evoked dilation is normalized back to the original baseline to represent the relative recovery/expansion

Statistical Unit: The mean percentage change from at least 9 measured vessels was average to generate a single representative data point for that individual mouse. Final statistical comparison was performed based on the number of mice.

1. **Quantification of BBB Leakage (Evans Blue and Albumin Immunofluorescence)**

ROI Definition and Image Processing (ImageJ, v1.8.0): To assess regional BBB permeability, brain regions (cortex, corpus callosum, Subcortex) were identified and navigated using the DAPI channel as a structural reference, in accordance with the Paxinos and Franklin mouse brain atlas. Using the DAPI channel as a guide, ROIs for specific brain regions were manually delineated. The ROI was then applied to the Evans Blue/Albumin channel for intensity measurement. A Rolling Ball algorithm (radius = 50 pixels) was applied to the leakage channels to remove non-specific background fluorescence. The Integrated Density was measured for the Evans Blue and Albumin channels within each ROI. To determine the fold-change in BBB leakage, the Integrated Density of the experimental groups was normalized to that of the Sham group within the same brain region.

Statistical unit: The relative values from all FOVs within a specific brain region per mouse were averaged. Final statistical comparisons were performed using the number of mice.

**Sample Size**

| **Assay**  ***in vitro*** | **Groups** | **Independent Experiments,**  **n** | **Exclusion/Dropout** | **Reason for Exclusion** |
| --- | --- | --- | --- | --- |
| NTA: EV size by time | EVs | 4 |  |  |
|  | cNGR-EVs | 4 |  |  |
| NTA:  EV size | EVs | 5 |  |  |
|  | cNGR-EVs | 5 |  |  |
|  | Cy3-EVs | 3 |  |  |
|  | Cy3-cNGR-EVs | 3 |  |  |
|  | FITC-cNGR-EVs | 3 |  |  |
| NTA:  EV size | Scr-EVs | 3 |  |  |
| WB: CD13 | HT29 | 4 |  |  |
|  | HT1080 | 4 |  |  |
|  | HBVP | 4 |  |  |
| CCK | Control | 3 |  |  |
|  | EVs | 3 |  |  |
|  | cNGR-EVs | 3 |  |  |
| Flow cytometry analysis | Control | 5 |  |  |
|  | Cy3-EVs | 5 |  |  |
|  | Cy3-Scr-EVs | 5 |  |  |
|  | Cy3-cNGR-EVs | 5 |  |  |
| ICC: Pericyte uptake EVs in vitro | Control | 6 |  |  |
|  | Cy3-EVs | 6 |  |  |
|  | Cy3-Scr-EVs | 6 |  |  |
|  | Cy3-cNGR-EVs | 6 |  |  |
| Pericyte contraction: HBVP Normoxia | Control | 6 |  |  |
|  | EVs | 6 |  |  |
|  | cNGR-EVs | 6 |  |  |
| Pericyte contraction : HBVP Hypoxia | Control | 7 |  |  |
|  | EVs | 7 |  |  |
|  | cNGR-EVs | 7 |  |  |
| Pericyte contraction : PC Normoxia | Control | 6 |  |  |
|  | EVs | 6 |  |  |
|  | Scr-EVs | 6 |  |  |
|  | cNGR-EVs | 6 |  |  |
| Pericyte contraction : PC Hypoxia | Control | 6 |  |  |
|  | EVs | 6 |  |  |
|  | Scr-EVs | 6 |  |  |
|  | cNGR-EVs | 6 |  |  |

| **Assay *ex vivo/ in vivo*** | **Group** | **Mice, n** | **Exclusions**  **/Dropouts** | **Reason for Exclusions**  **/Dropouts** |
| --- | --- | --- | --- | --- |
| Ex vivo organ Near-Infrared Imaging | Control | 4 |  |  |
|  | Cy7-EVs | 4 |  |  |
|  | Cy7-Scr-EVs | 4 |  |  |
|  | Cy7-cNGR-EVs | 4 |  |  |
| ICC: Regional pericyte EV uptake in brain slices | Control | 5 |  |  |
|  | Cy3-EVs | 5 |  |  |
|  | Cy3-Scr-EVs | 5 |  |  |
|  | Cy3-cNGR-EVs | 5 |  |  |
| Eight-arm radial maze | Sham | 8 |  |  |
|  | BCAS | 8 |  |  |
|  | BCAS+EVs | 7 | 1 Exclusion/ 1 Dropout | 1 accidental death before testing 1 accidental death on Day 6 of testing |
|  | BCAS+cNGR-EVs | 8 |  |  |
| Elevated plus maze | Sham | 9 |  |  |
|  | BCAS | 7 | 2 Exclusions | 2 accidental death |
|  | BCAS+EVs | 8 | 1 Exclusion | 1 accidental death |
|  | BCAS+cNGR-EVs | 9 |  |  |
| Myelin G-ratio quantification | Sham | 4 |  |  |
|  | BCAS | 4 |  |  |
|  | BCAS+EVs | 4 |  |  |
|  | BCAS+cNGR-EVs | 4 |  |  |
| WB: MBP/Neurofilament | Sham | 4 |  |  |
|  | BCAS | 5 |  |  |
|  | BCAS+EVs | 5 |  |  |
|  | BCAS+cNGR-EVs | 5 |  |  |
| Blood flow assessment | Sham | 6 |  |  |
|  | BCAS | 6 |  |  |
|  | BCAS+EVs | 6 | 1 Dropout | 1 accidental death on Day 7 of assessment |
|  | BCAS+cNGR-EVs | 8 | 1 Dropout | 1 accidental death on Day 30 of assessment |
| ICC: Pericyte coverage | Sham | 5 |  |  |
|  | BCAS | 5 |  |  |
|  | BCAS+EVs | 5 |  |  |
|  | BCAS+cNGR-EVs | 5 |  |  |
| Quantification of capillary diameter changes: NA | Sham | 6 |  |  |
|  | BCAS | 6 |  |  |
|  | BCAS+EVs | 5 | 1 Exclusion | 1 technical failure during the slicing procedure |
|  | BCAS+cNGR-EVs | 6 |  |  |
| Quantification of capillary diameter changes: Glu | Sham | 3 | 3 Exclusions | Technical artifacts  (e.g., focal drift or cessation of microcirculation during sequential drug application). |
|  | BCAS | 3 | 3 Exclusions | Technical artifacts  (e.g., focal drift or cessation of microcirculation during sequential drug application). |
|  | BCAS+EVs | 5 | 1 Exclusion | 1 technical failure during the slicing procedure |
|  | BCAS+cNGR-EVs | 4 | 2 Exclusions | Technical artifacts (e.g., focal drift or cessation of microcirculation during sequential drug application) |
| Quantification of BBB Leakage: Evans Blue | Sham | 5 |  |  |
|  | BCAS | 5 |  |  |
|  | BCAS+EVs | 5 |  |  |
|  | BCAS+cNGR-EVs | 5 |  |  |
| Quantification of BBB Leakage: Albumin | Sham | 4 |  |  |
|  | BCAS | 5 |  |  |
|  | BCAS+EVs | 4 |  |  |
|  | BCAS+cNGR-EVs | 4 |  |  |
| snRNA-seq | Sham | 3 |  |  |
|  | BCAS | 3 |  |  |
|  | BCAS+EVs | 2 | 1 Exclusion | 1 accidental death |
|  | BCAS+cNGR-EVs | 4 |  |  |
