# Supplementary material for: Engineered Pericyte‐Targeted Extracellular Vesicles Protect Against Hypoperfusion‐Induced Cognitive Impairment and Vascular Demyelination
Source: J Extracell Vesicles. 2026 Jun 1;15(6):e70319. doi: 10.1002/jev2.70319 (PMC13240518; doi:10.1002/jev2.70319)
Supplement: Supplementary file 2 — Supplementary Figures S1‐S10: jev270319‐sup‐0002‐SuppMat.docx Supplemented materials and methods [file JEV2-15-e70319-s002.docx]

**Engineered Pericyte-targeted Extracellular Vesicles Protect against Hypoperfusion-induced Cognitive Impairment and Vascular Demyelination**

Weiwei Shen^1†^, Weishi Liu^1†^, Min Guo^1†^, Tongyao You^1^, Yingzhe Wang^1^, Tiansiyu Wen^1^, Suzhen Liang^1^, Xiaodi Xie^1^, Yanfeng Jiang^3^, Qiang Dong^1,2^*, Jintai Yu^1,2^*, Mei Cui^1,2^*

^1^Department of Neurology and National Center for Neurological Disorders, Huashan Hospital, Fudan University; Shanghai 200040, China.

^2^State Key Laboratory of Brain Function and Disorders and MOE Frontiers Center for Brain Science, Shanghai Medical College, Fudan University; Shanghai 200032, China.

^3^Human Phenome Institute, Zhangjiang Fudan International Innovation Center, Fudan University; Shanghai 201203, China.

*Corresponding author. Email: [dong_qiang@fudan.edu.cn](mailto:dong_qiang@fudan.edu.cn) (Q.D.); [jintai_yu@fudan.edu.cn](mailto:jintai_yu@fudan.edu.cn) (J.Y.); [cuimei@fudan.edu.cn](mailto:cuimei@fudan.edu.cn) (M.C.)

^†^These authors contributed equally to this work.

**The PDF file includes:**

Supplemented Materials and methods

Figs. S1 to S10

**Supplemented** **Materials and methods**

**Molecular docking**

Molecular docking of cNGR peptide to human CD13 (hCD13; PDB: 4FYR) was performed using AutoDock-GPU (v4.2.6). The cNGR structure (ChemDraw-derived, RDKit-minimized) was docked against the hCD13 crystal structure (retaining Zn²⁺ cofactor, water molecules removed, hydrogens added). The substrate binding site of hCD13 was used as the docking site (grid center: x= 107.851, y= 17.319, z= 22.494 in Å; grid dimensions: 50 Å × 40 Å × 40 Å）. Flexible ligand docking employed the Lamarckian Genetic Algorithm (100 runs) with empirical free energy scoring. The top-scoring conformation was visualized in Discovery Studio (v18.1) and PyMOL (v2.5.0).

**Cell viability measurement and carbachol contraction test**

Cell viability measurement: To assess the effects of cNGR-EVs on pericyte viability, HBVP were seeded in 96-well plates (5000 cells/well) and treated with either control EVs， Scr-EVs, or cNGR-EVs (45 µg/mL) for 24 h under standard culture conditions. Viability was quantified using CCK-8 reagent (Sangon Biotech, #E606335) per the manufacturer’s protocol. Absorbance (450 nm) was measured after 1 h incubation at 37℃. All samples were run in triplicate.

Carbachol contraction test: HBVP/Pericytes were seeded in confocal dishes and treated with either control EVs, Scr-EVs, or cNGR-EVs (45 µg/mL) for 24 h under normoxia (21% O_2_). For hypoxia-challenged conditions, EVs-treated cells were cultured for 48 h in reduced oxygen (2% O_2_). Contractility was induced with 1 µM carbachol (Sigma-Aldrich) and monitored for 10 min via time-lapse imaging (Olympus SpinSR; 40× air objective). The percentage change in surface area during contraction was quantified across five random fields using ImageJ software.

**Near-infrared fluorescence (NIRF) imaging**

At 2 h post-intranasal administration of Cy7-labeled EVs (including Cy7-EVs, Cy7-Scr-EVs and Cy7-cNGR-EVs, , n=4 mice/group), mice were sacrificed and major organs (brain, heart, lungs, spleen, kidneys, liver, and gastrointestinal tract) were immediately subjected to NIRF imaging using the VISQUE In Vivo Smart-LF system (Vieworks) with CleVue software (v3.1.3.2054) for Cy7 signal quantification.

**Blood brain barrier (BBB) permeability examination**

On day 3 post-procedure, mice received intraperitoneal Evans blue injection (2% solution, 10 mL/kg; Sigma-Aldrich). Successful administration was confirmed by visible dye in peripheral tissues (ears, paws, tail). After 2 hours, mice were anesthetized and processed for tissue collection. Serial 30-µm coronal sections were imaged using an Olympus Slideview VS120 system with OlyVIA software (v2.9.1). BBB permeability was quantified by measuring the integrated density of Evans blue extravasation in Ctx, CC and SCtx using ImageJ software. For complementary assessment, albumin extravasation was evaluated by immunofluorescence staining with anti-albumin antibody (1:200, Bethyl Laboratories #A90-134A) and appropriate secondary antibodies.

**Processing of single-nucleus RNA sequencing (snRNA-seq) data**

Nucleus suspension preparation: Brains were dissected from Sham, BCAS, BCAS+EVs and BCAS+cNGR-EVs groups, perfused with cold PBS buffer, and flash frozen in liquid nitrogen. Nuclei were isolated from approximately 100 mg of tissue. The tissue was minced and homogenized on ice in 2 mL of pre-chilled Homogenization Buffer using a mechanical homogenizer with 10-15 strokes. The homogenate was sequentially filtered through 70 µm and 40 µm cell strainers. The filtrate was centrifuged at 500 × *g* for 5 min at 4°C, and the pellet was washed twice with Wash Buffer. The final nuclei pellet was resuspended in Blocking Buffer. Nuclear integrity and background contamination were assessed by microscopy.

Library preparation: The nucleus suspension was stained with 0.4% trypan blue or AO/PI. Nucleus integrity was assessed under a microscope. Samples passing quality control were used for subsequent library preparation. The suspensions, oil, and barcoded beads were loaded onto a microfluidic chip. Water-in-oil emulsions were generated using the DNBelab C-TaiM instrument, enabling cell lysis and mRNA capture by bead-conjugated oligonucleotides within the droplets. Captured mRNA was reverse transcribed into cDNA inside the droplets. Droplets were broken using a demulsification reagent. The mixture was incubated at room temperature and centrifuged to separate the aqueous phase. cDNA products were extracted and purified using magnetic beads. Purified cDNA was amplified via PCR. The resulting product was cleaned up for subsequent steps. Short oligonucleotides released during demulsification were amplified by PCR, indexed with sample-specific barcodes, and purified to construct the oligo library. The amplified cDNA was fragmented, end-repaired, and A-tailed. Adaptors were ligated to the fragments, followed by PCR amplification to construct the final cDNA library. Each intermediate product was purified after the respective reaction. Both cDNA and oligo libraries were denatured into single-stranded DNA and circularized. Any remaining linear DNA molecules were digested. Single-stranded circular DNA was amplified via rolling circle amplification to produce DNA nanoballs (DNBs). DNBs were loaded into a patterned nanoarray flow cell and sequenced using combinatorial probe-anchor synthesis (cPAS) technology.

snRNA-seq data processing: snRNA-seq reads were aligned to the mm10 genome using CellRanger (v7.2.0) with default settings. The resulting data for each sample were processed in Seurat (v5.1.0), retaining genes detected in at least three cells and cells expressing a minimum of 200 genes. Cells were further filtered to exclude those with fewer than 300 or more than 10,000 detected genes, or with mitochondrial gene content exceeding 10%. After quality control, 202,182 high-quality cells remained. Gene expression values were normalized using Seurat’s default method, and the top 2,000 highly variable genes (HVGs) were identified via the FindVariableFeatures function, followed by data scaling. Principal component analysis (PCA) was applied to the processed matrix for dimensionality reduction.

To mitigate batch effects between samples, integration was performed using the IntegrateLayers function in Seurat with the CCA Integration method, based on the top 30 principal components.

**Supplementary Figures**

**
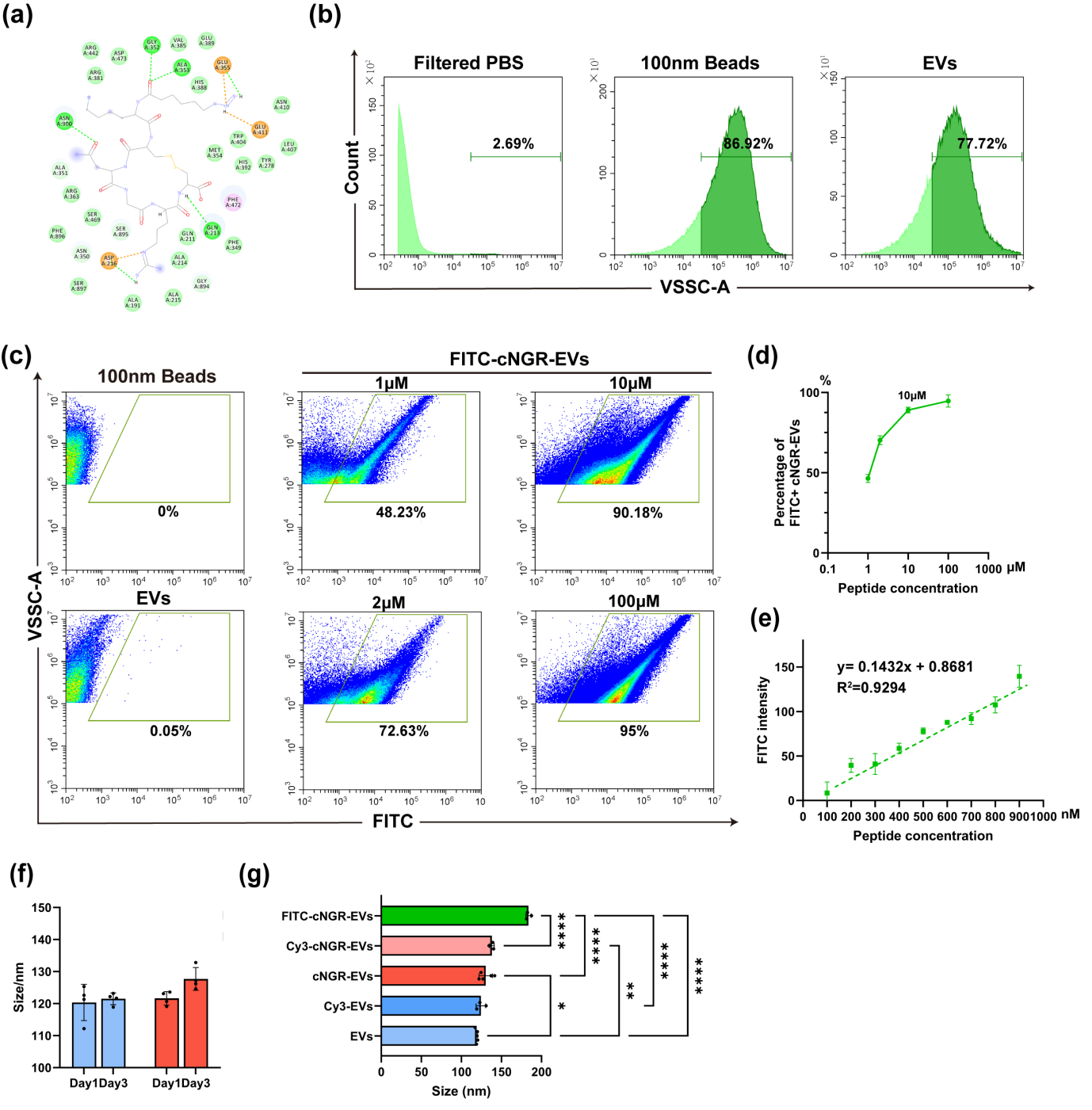
**

**Figure S1. Optimization of sEV-peptide conjugation. (a)** Computational model of cNGR-hCD13 interactions. Green dashed lines indicate hydrogen bonds; orange dashed lines indicate salt bridges/charge interactions; light green residues indicate Van der Waals contacts; pink residues indicate hydrophobic interactions. (**b)** Gating strategy for single-EV flow cytometry. EVs were distinguished from background (VSSC < 10^4^) using 100-nm reference beads. **(c)** Single-EV flow cytometry analysis of FITC-cNGR titration experiment. DBCO-EVs (100 μg) were incubated with FITC-cNGR at 1, 2, 10, and 100 μM, followed by purification to remove unreacted peptides. The percentage of FITC-positive cNGR-EVs increased in a concentration-dependent manner (~48%, 72%, 90%, and 95%, respectively). **(d)** Titration curve indicating 10 μM as the working concentration, achieving near-saturation (90%). **(e)** Quantification of cNGR conjugation using a standard curve of free FITC-cNGR (0.1–0.9 μM). The cNGR concentration in purified FITC-cNGR-EVs (1μg/μL, intensity 29.5) was estimated to be ~0.2 μM. The FITC-cNGR-EVs particle concentration, determined by nanoparticle tracking analysis (NTA), was 6.6×10^10^ particles/mL. Peptide density=Total cNGR molecules/Total EV particles≈1.8 × 10³molecules/EV. **(f)** Size stability of unmodified EVs and cNGR-EVs during storage (day 1 vs. day 3; n = 4 per group; paired t-test). **(g)** Size distribution of EV variants measured by NTA, including control EVs, Cy3-EVs, cNGR-EVs, Cy3-cNGR-EVs and FITC-cNGR-EVs. EVs/cNGR-EVs: n=5 biological replicates per group, Cy3-EVs/Cy3-cNGR-EVs/FITC-cNGR-EVs: n=3 biological replicates per group. One-way ANOVA with Tukey’s test. Data are presented as mean ± SD. Detailed sample values and statistical information are provided in the source data Table S5. **P* < 0.05, ***P* < 0.01, ****P* < 0.001, *****P* < 0.0001.

**
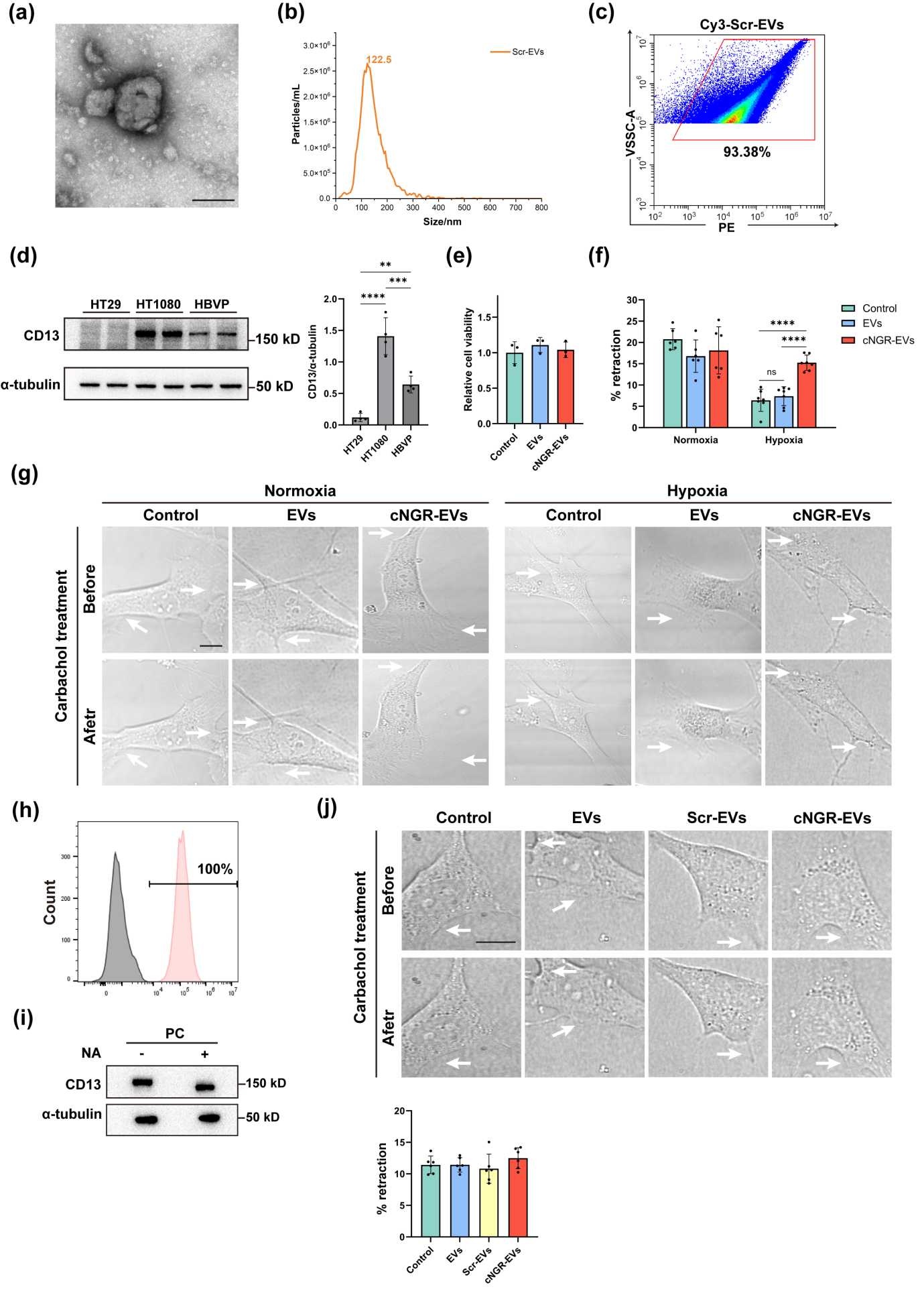
**

**Figure S2. Characterization of Scr-EVs and effects of EV variants on pericyte viability and contractility.** **(a)** Representative TEM image of Scr-EVs. Scale bar: 100 nm. **(b)** Representative size distribution profile of Scr-EVs measured by nanoparticle tracking analysis (NTA). **(c)** Labeling efficiency of Scr-EVs analyzed by Single-EV flow cytometry. **(d)** Immunoblot analysis of CD13 expression in HT29, HT1080, HBVP lysates, with corresponding quantification. n=4 independent experiments per group, One-way ANOVA with Tukey's multiple comparisons test. **(e)** Viability of HBVP under normoxia assessed by the CCK8 assay. n=3 independent experiments per group, Kruskal-Wallis test, *P=*0.5107. **(f)** Quantification of carbachol-induced changes in HBVP cell area across treatment groups under normoxia and hypoxia. Normoxia: n=6 independent experiments per group, Welch's ANOVA test: *P*=0.1533. Hypoxia: n=7 independent experiments per group, One-way ANOVA with Tukey’s test. **(g)** Representative images of HBVP morphology before and 10 min after carbachol treatment under normoxia and hypoxia. White arrows indicate retraction of pericyte processes within the field of view. Scale bar: 20 μm. **(h)** Flow cytometric quantification of CD13 isoform expression in pericytes. **(i)** Immunoblot analysis of CD13 expression in pericyte with (+) or without (-) neuraminidase (NA) treatment. NA induces a downward shift of the CD13 band. α-tubulin was used as a loading control. **(j)** Representative images and quantification of carbachol-induced contraction in pericytes under normoxia. White arrows indicate processes retraction. Scale bar: 10 μm. n=6 independent experiments per group, one-way ANOVA with Tukey’s test, *P*=0.4058. Data are presented as mean ± SD. Detailed statistical information is provided in the source data Table S6. ***P* < 0.01, ****P* < 0.001, *****P* < 0.0001. Abbreviation: NA, neuraminidase; Nor, normoxia; Hypo, hypoxia.

**
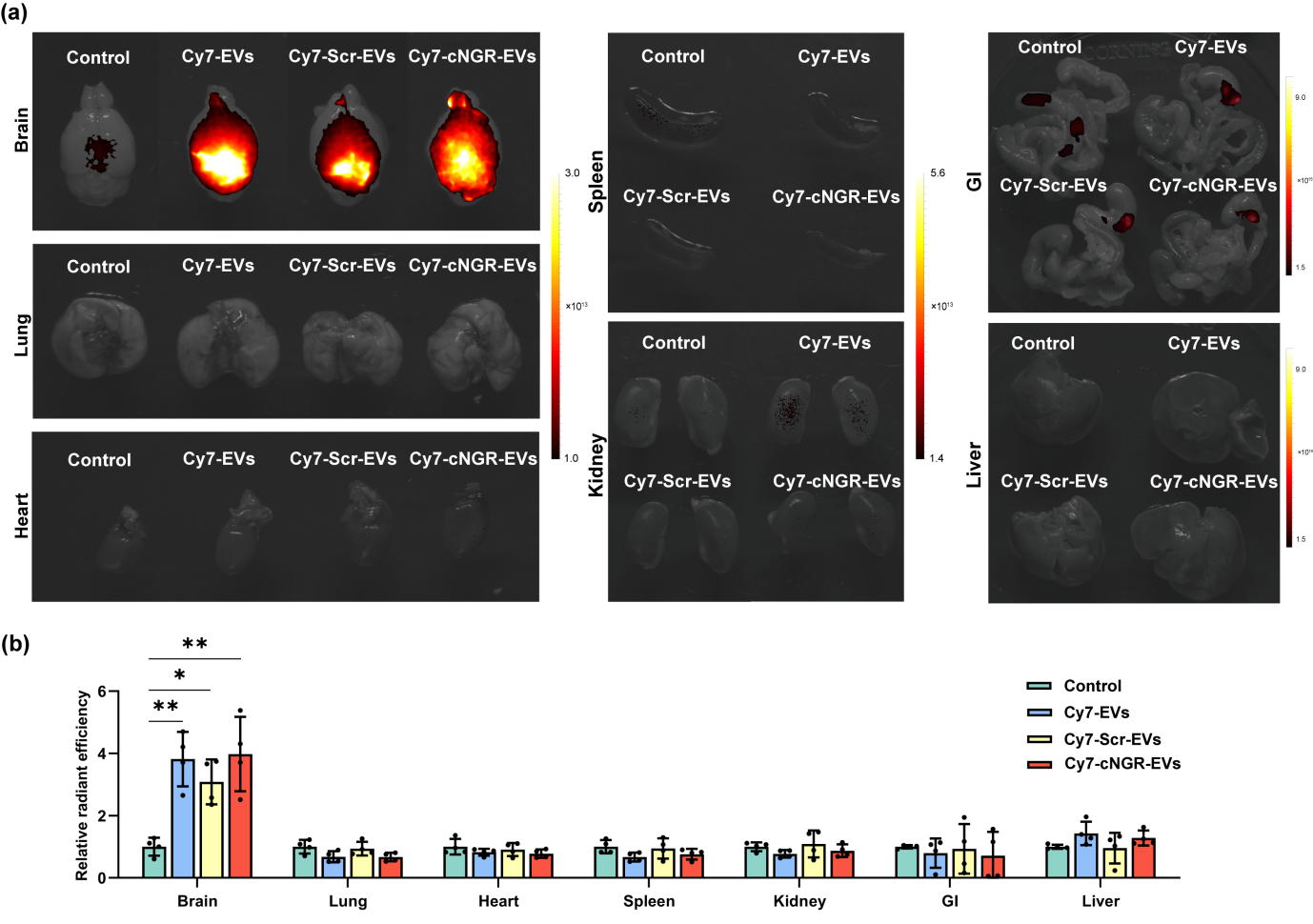
**

**Figure S3. Biodistribution of intranasally administered cNGR-EVs**. **(a)** Representative *in vivo* NIRF images showing the biodistribution of Cy7-labelled EVs variants, including Cy7-EVs, Cy7-Scr-EVs, Cy7-cNGR-EVs, at 2 hours after intranasal administration. **(b)** Quantification of Cy7 radiant efficiency in organs, normalized to the PBS control group. Data are presented as mean ± SD, n=4 mice per group. Detailed statistical information is provided in the source data Table S7. **P* < 0.05，***P* < 0.01. Abbreviation: GI, gastrointestinal tract.

**
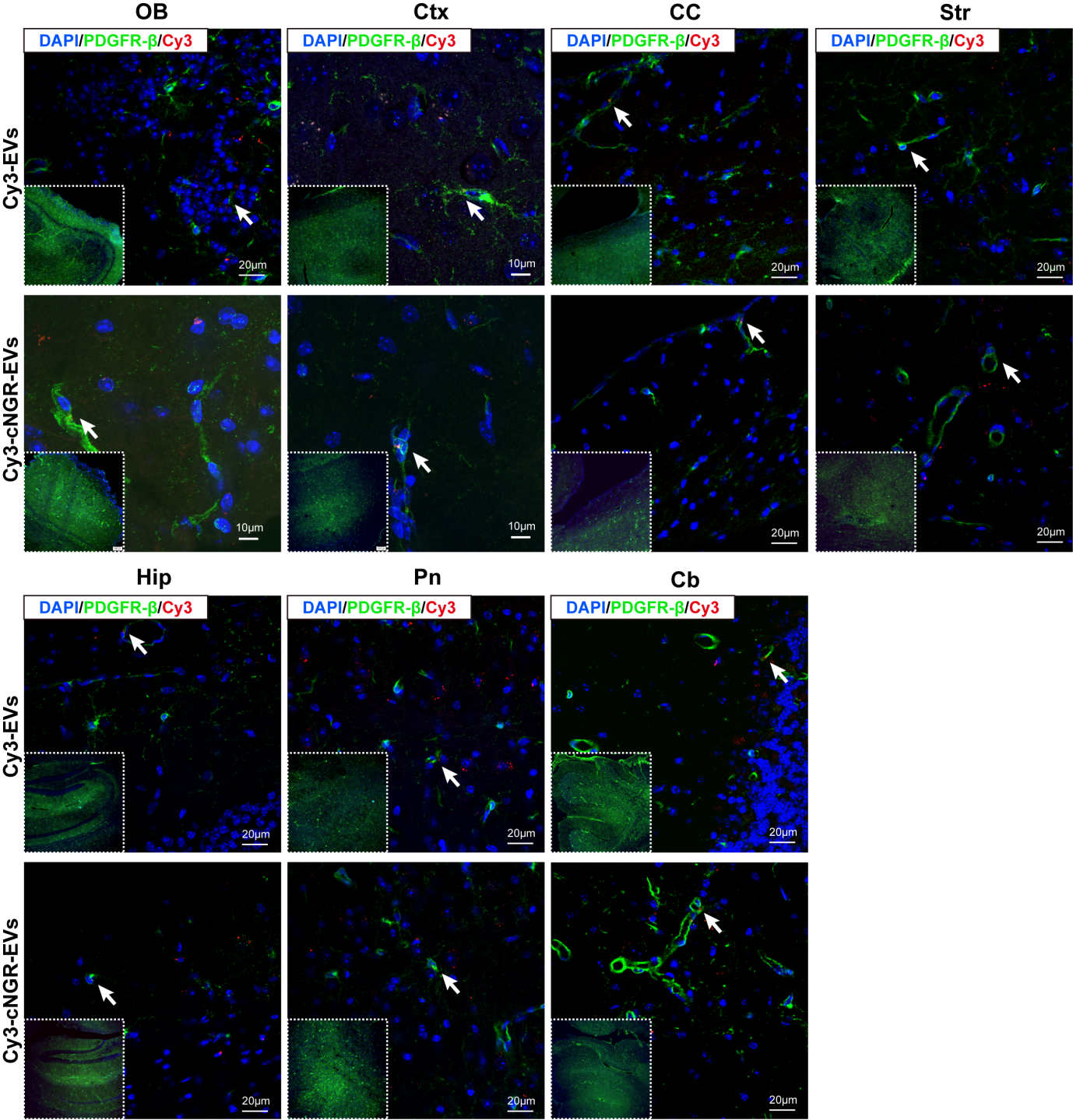
**

**Figure S4. Low-magnification fluorescence images of EV distribution in the brain.** Representative confocal images showing the distribution of Cy3-EVs and Cy3-cNGR-EVs (red) in various brain regions, with lower-magnification images identifying the anatomical regions (dashed box). The white arrows indicate the specific cells that are presented at higher magnification in Figure 3a. Abbreviations: OB, olfactory bulb; Ctx, cortex; CC, corpus callosum; Str, striatum; Hip, hippocampus; Pn, pons; Cb, cerebellum.

**
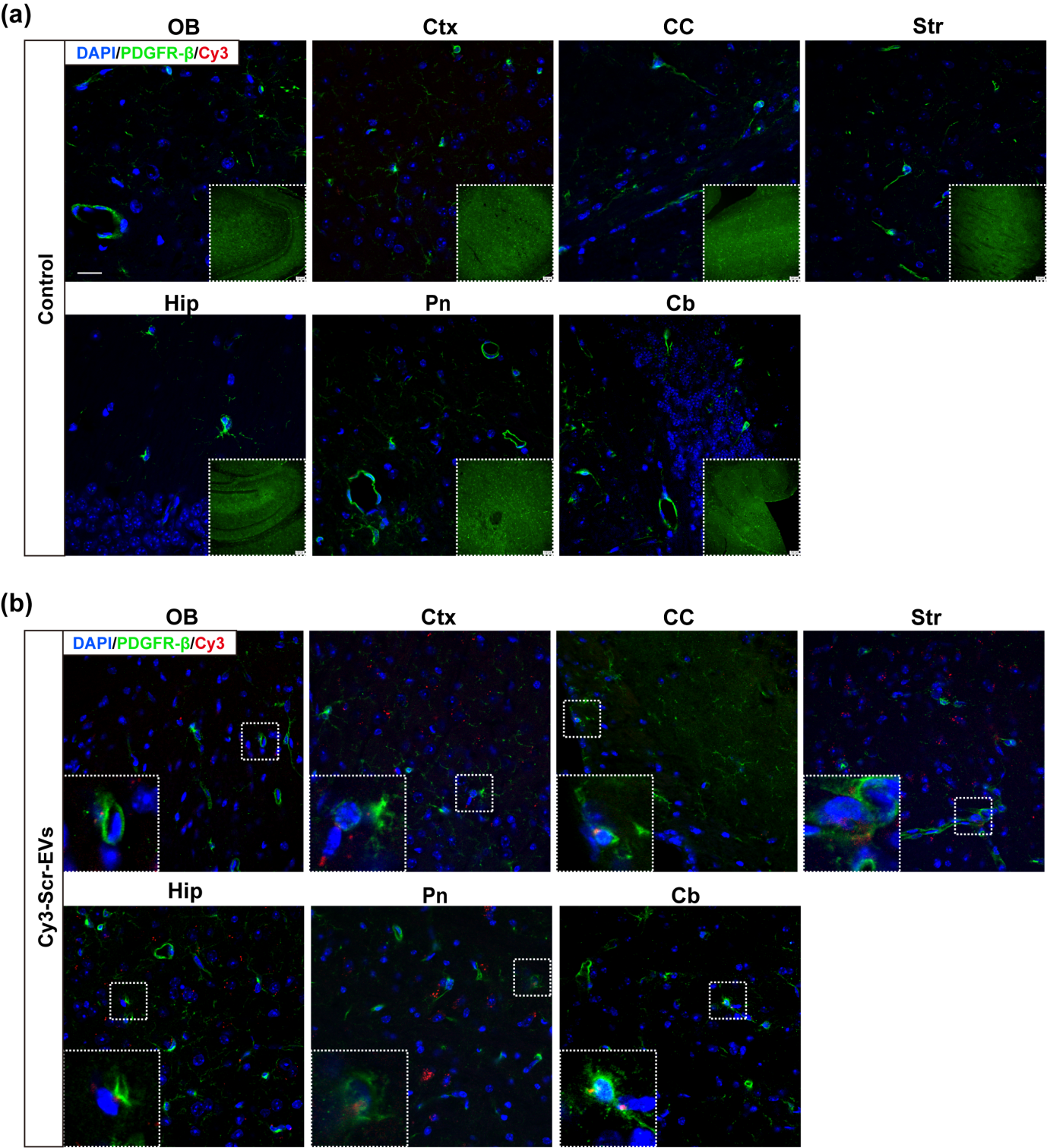
**

**Figure S5. Colocalization of Pericyte and EVs in control and the Scr-EVs-treated mice. (a)** Representative confocal microscopy showing negligible Cy3 fluorescence in the brain of PBS-treated control mice, with lower-magnification images identifying the anatomical regions (dashed box). Scale bar: 20 μm. **(b)** Representative confocal microscopy images (dashed box) of Scr-EV uptake (Cy3^+^, red) by pericyte (PDGFRβ^+^, green) in the brain of Scr-EVs treated mice. Scale bar: 20 μm. Abbreviations: OB, olfactory bulb; Ctx, cortex; CC, corpus callosum; Str, striatum; Hip, hippocampus; Pn, pons; Cb, cerebellum.

**
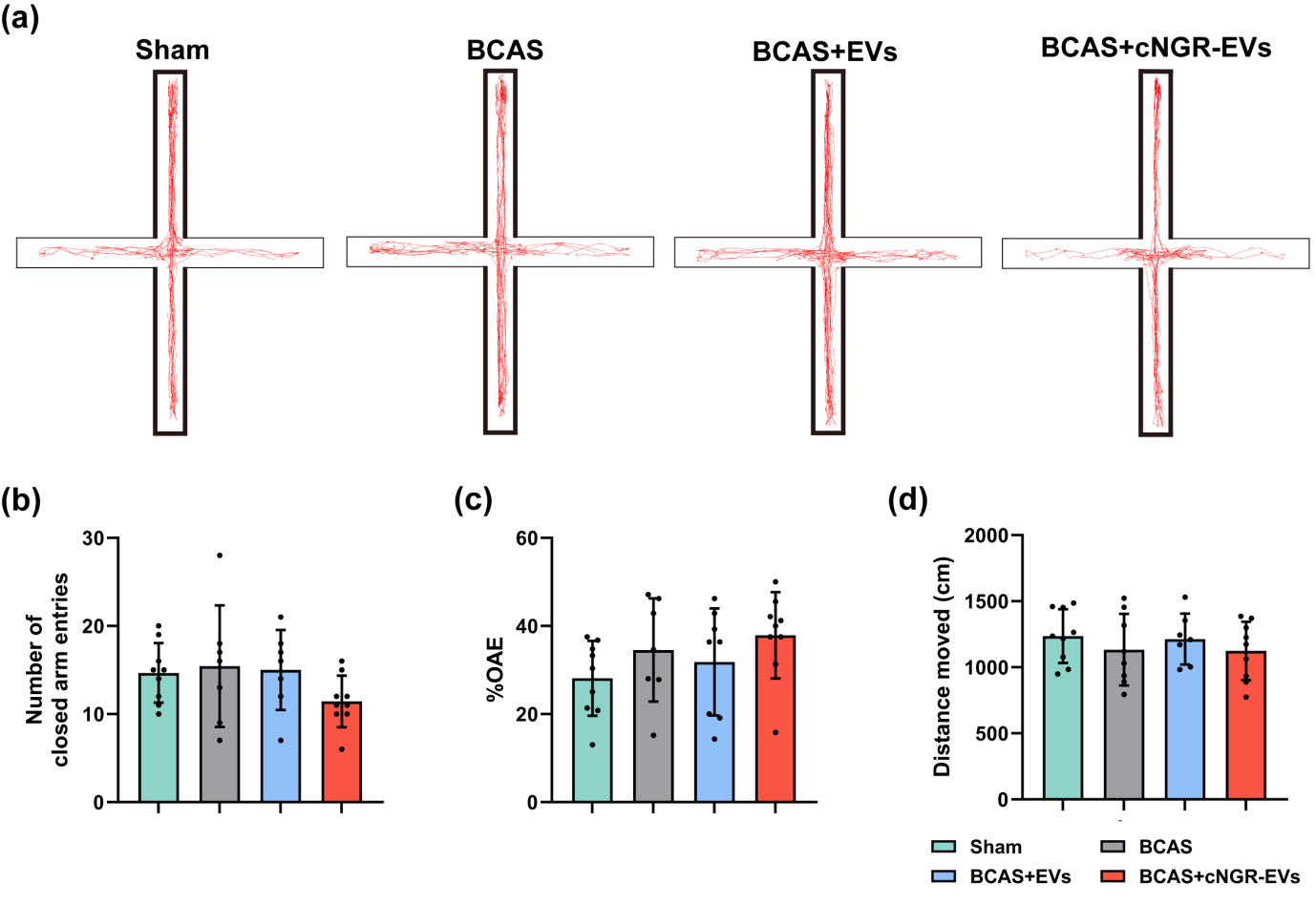
**

**Figure S6. Effects of cNGR-EVs on locomotor activity and anxiety-related behaviors following BCAS. (a)** Representative elevated plus maze movement trajectories across sham-operated (Sham), BCAS-operated (BCAS), negative control EVs-treated (BCAS+EVs) and cNGR-EVs-treated (BCAS+cNGR-EVs) mice on 30 days post-operation. Closed arms are indicated (bold lines). The BCAS group exhibited increased exploration of open arms compared to other groups. **(b-d)** Quantitative analysis of closed arm entries **(b)**, percentage of open arm entries (%OAE) **(c)** and total distance moved **(d)**. Data are presented as mean ± SD, Sham: n=9 mice, BCAS: n=7 mice, BCAS+EVs: n=8 mice, BCAS+cNGR-EVs: n=9 mice. No significant differences were detected between groups. Detailed statistical information are provided in the source data Table S8.

**
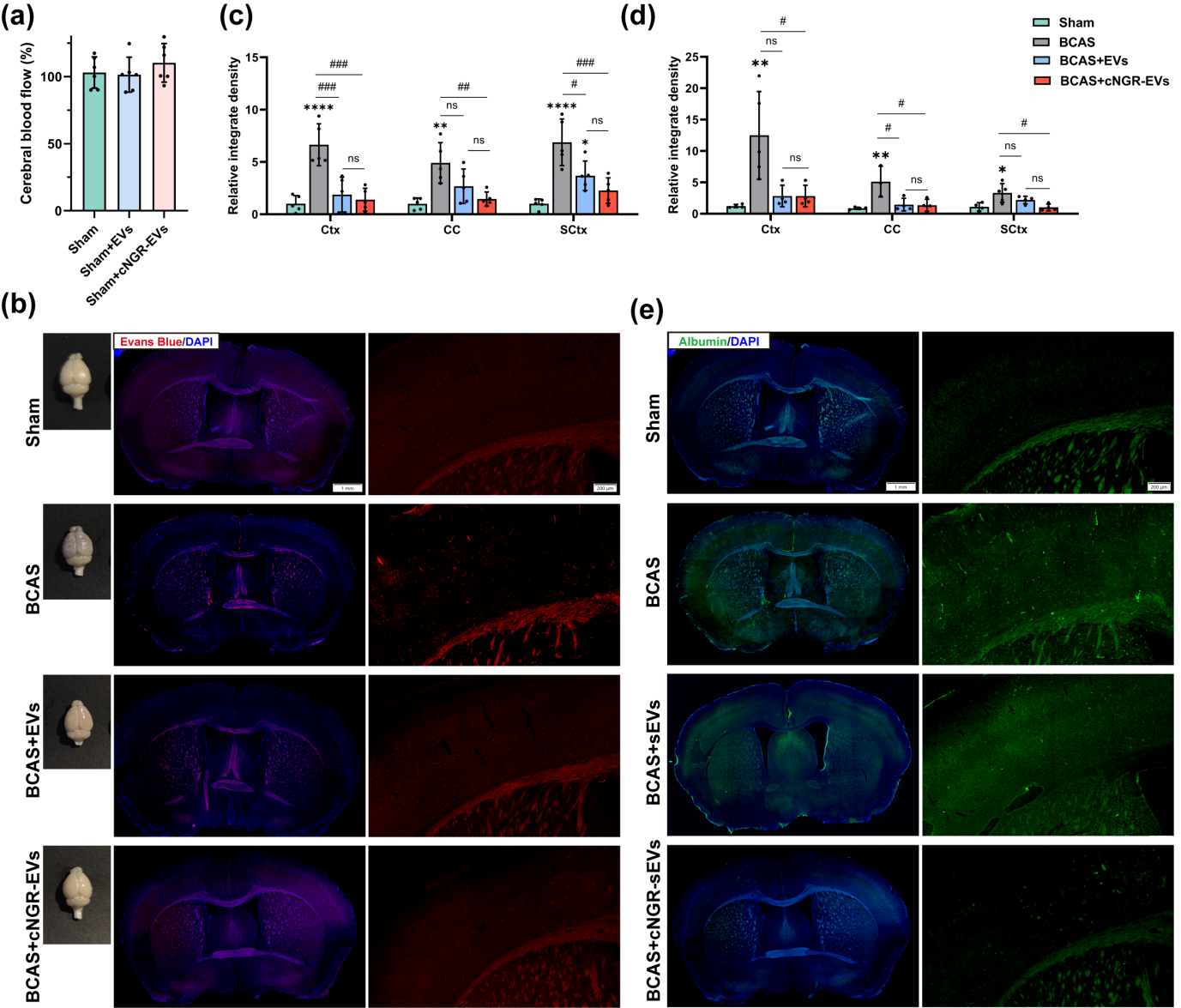
**

**Figure S7. Effects of cNGR-EVs on BBB integrity** **following BCAS.** **(a)** Quantitative of CBF changes (% of preoperative baseline) across groups on postoperative day 3. n=6 mice per group, one-way ANOVA with Tukey’s test. **(b)** Evans Blue extravasation assay assessing BBB permeability on postoperative day 3. Scale bar: 1 mm. **(c)** Quantitative analysis of Evans Blue fluorescence intensity in the cortex (Ctx), corpus callosum (CC), and subcortical regions (Stx), normalized to the Sham group. n=5 mice per group, one-way ANOVA with Tukey’s test. **(d)** Quantitative analysis of albumin fluorescence intensity in Ctx, CC, and Stx, normalized to the Sham group. Sham/BCAS+EVs/BCAS+cNGR-EVs: n=4 mice/group, BCAS: n=5 mice. One-way ANOVA with Tukey’s test. **(e)** Representative immunofluorescence images of albumin (green) in brain sections across groups on postoperative day 3. Scale bar: 1 mm. Data are presented as mean ± SD. Detailed statistical information are provided in Table S9. Significance levels: **P* < 0.05, ***P* < 0.01, *****P* < 0.0001 vs. Sham; ^#^*P* < 0.05, ^##^*P* < 0.01, ^###^*P* < 0.001.

**
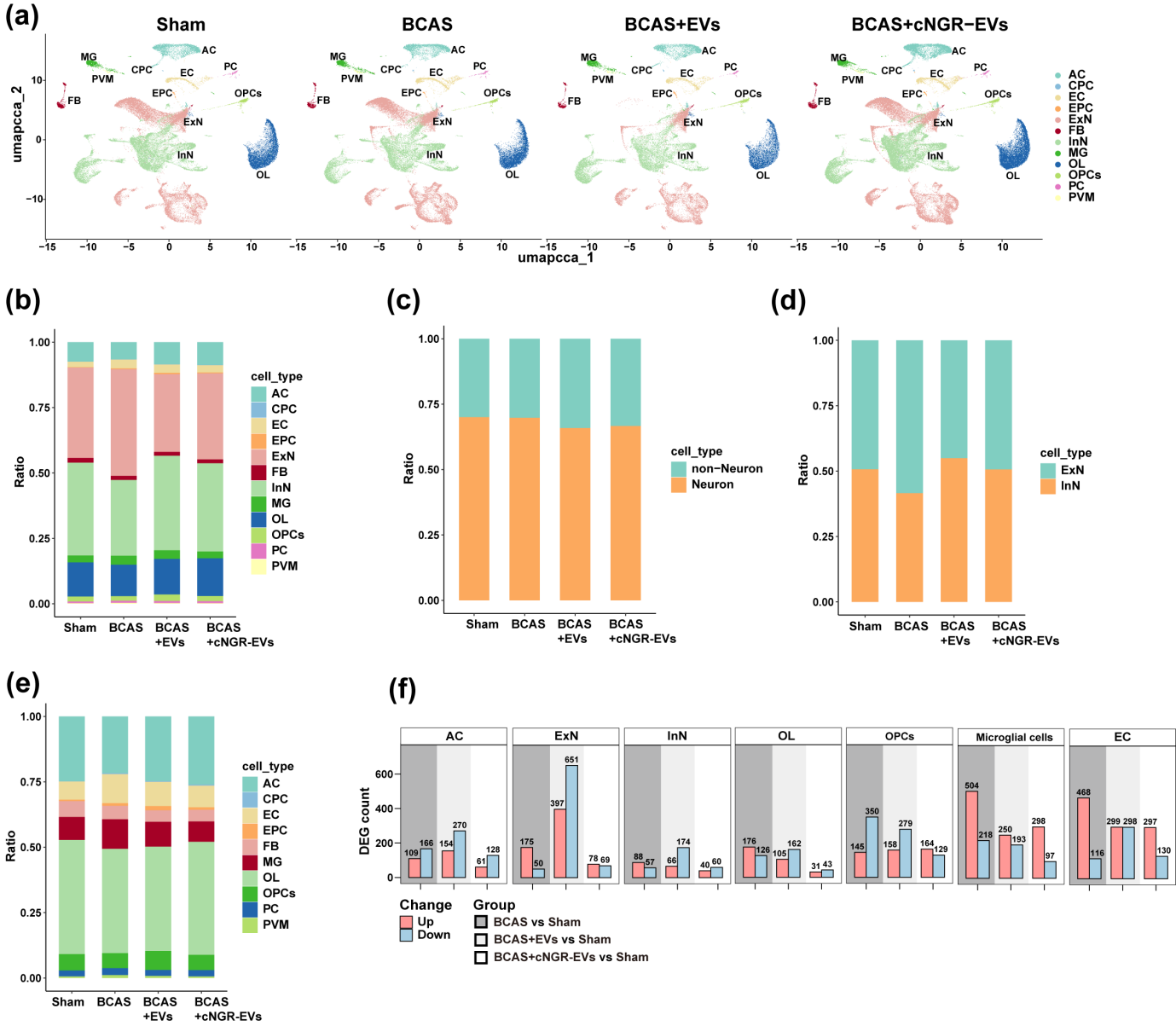
**

**Figure S8. Differential cell cluster abundance and cluster-specific transcriptional changes following BCAS.** **(a)** UMAP plots of single-cell RNA-seq data across four groups. **(b-e)** Stacked bar plots depicting the relative abundance of all cell clusters **(b)**, neuron and non-neuron populations **(c)**, excitatory and inhibitory neuronal subtypes **(d)**, and other non-neuronal cell types **(e)** across groups. **(f)** Bar plot summarizing numbers of DEGs in astrocytes, excitatory neurons, inhibitory neurons, oligodendrocytes, OPCs, microglia and endothelial cells across groups. Abbreviations: ExN, excitatory neurons; InN, inhibitory neurons; OL, oligodendrocytes; AC, astrocytes; MG, microglia; OPCs, oligodendrocyte progenitor cells; EC, endothelial cells; PC, pericytes; CPC, choroid plexus cells; EPC, ependymocytes ; FB, fibroblasts; PVM, perivascular macrophages. Detailed breakdown of cell numbers categorized by specific cell types is provided in the source data Table S10.

**
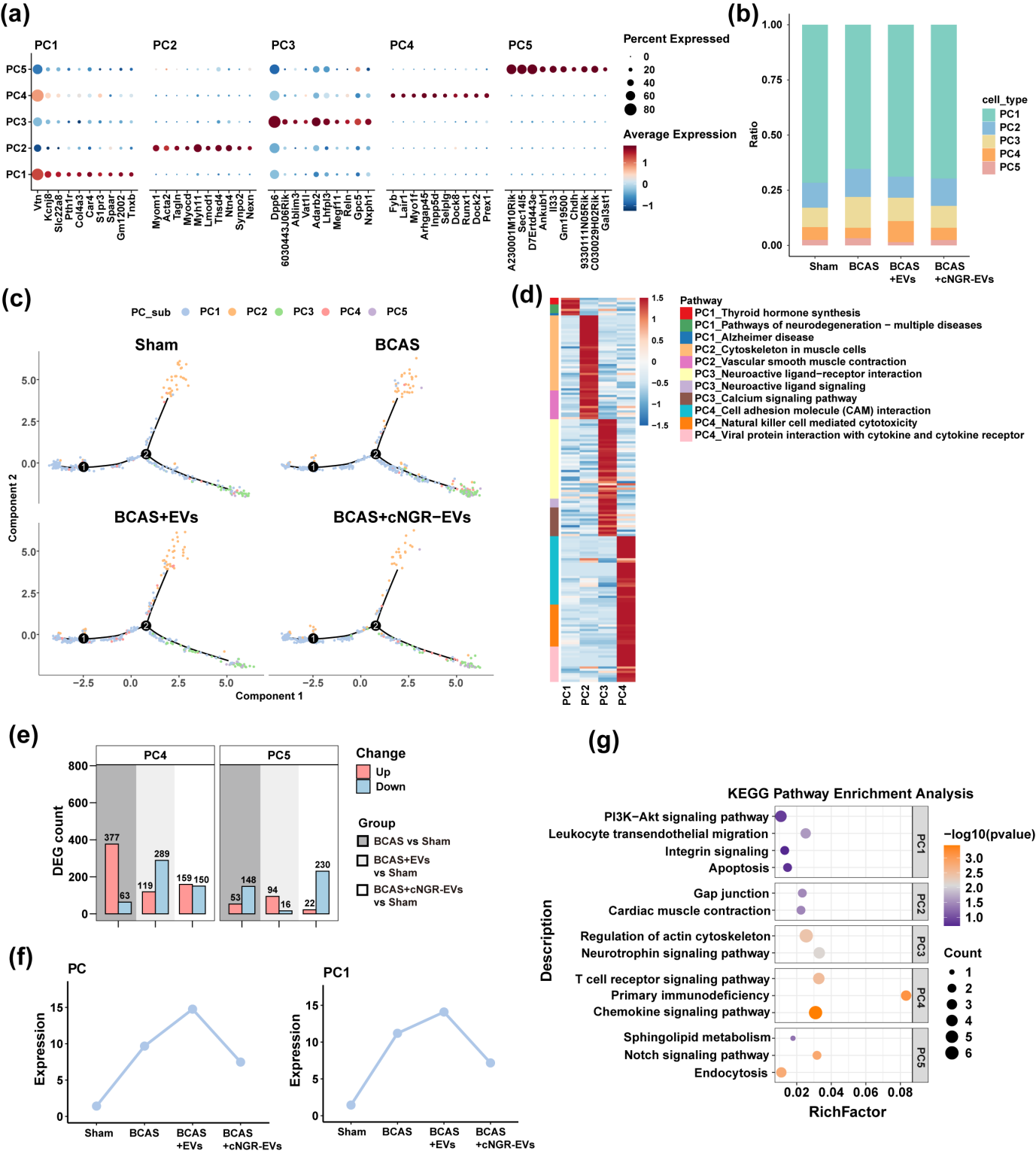
**

**Figure S9. Heterogeneity analysis of pericyte subclusters.** **(a)** Proportion and average expression of marker genes across pericyte subclusters. **(b)** Stacked bar plots depicting the relative ratio of each pericyte subcluster per group. **(c)** Pseudotime trajectory of pericyte subclusters across groups. **(d)** KEGG pathway enrichment analysis of upregulated genes in pericyte subclusters. **(e)** Bar plots showing the number of DEGs in PC4 and PC5, from BCAS vs. Sham, BCAS+EVs vs. Sham and BCAS+cNGR-EVs vs. Sham comparisons. **(f)** Line plots showing the expression dynamics of *Ttr* across conditions in pericyte and PC1 subcluster. **(g)** Bubble plots displaying significantly enriched KEGG pathways for genes reversed by cNGR-EVs treatment across subclusters. Abbreviations: PC, pericytes.


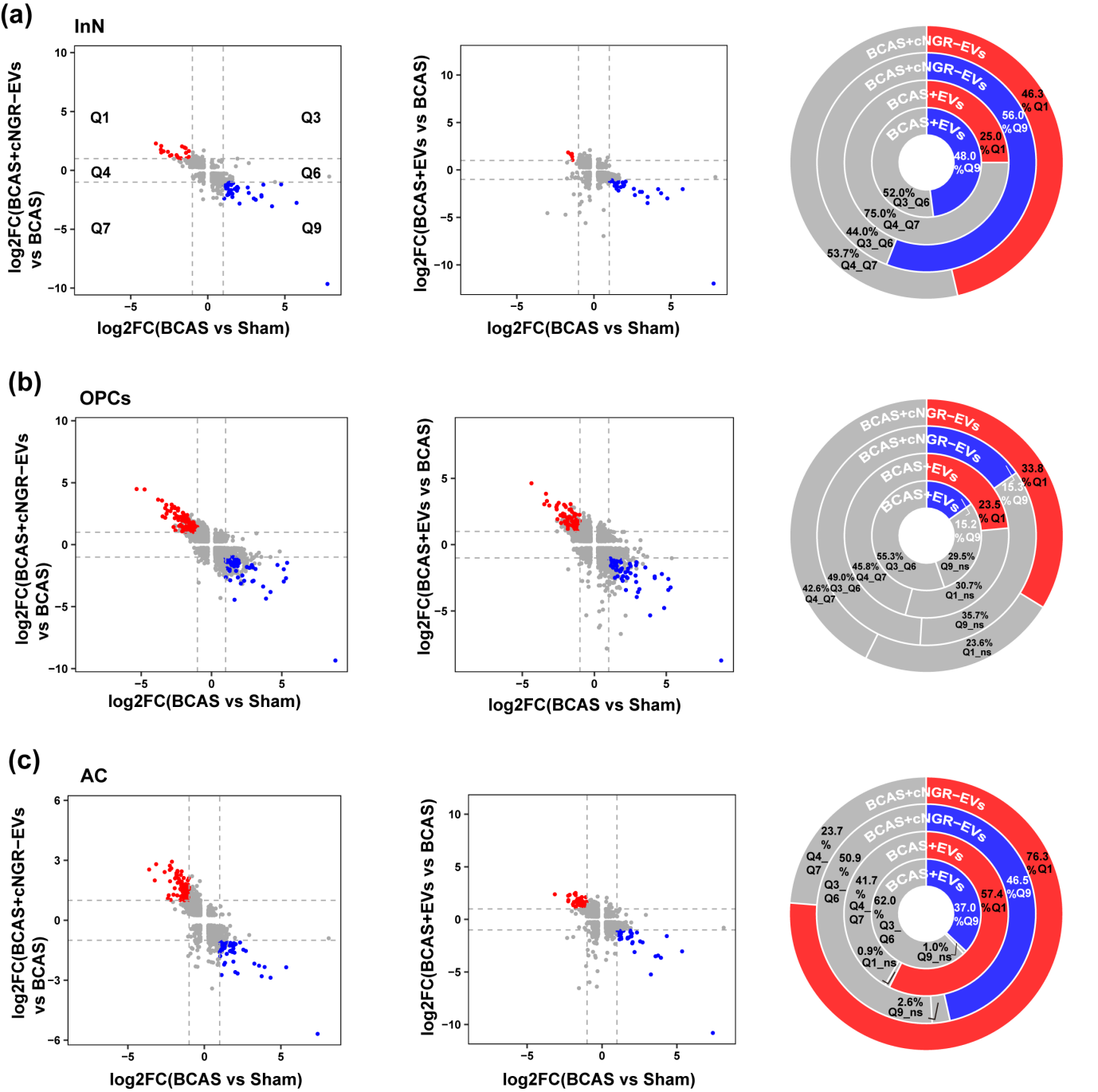


**Figure S10. Transcriptomic preservation in inhibitory neurons, OPCs and astrocytes following cNGR-EVs treatment.** Nine-quadrant plots (left) comparing transcriptomic changes (log2 Fold Change) induced by BCAS (x-axis) versus those induced by treatment (y-axis; BCAS+cNGR-EVs or BCAS+EVs) in inhibitory neurons **(a)**, OPCs **(b)** and astrocytes **(c)**. Genes in Q1 (downregulated by BCAS but upregulated by cNGR-EVs treatment) and Q9 (upregulated by BCAS but downregulated by cNGR-EVs treatment) are defined as reversed DEGs (highlighted in red and blue, respectively). Nested pie charts (right) show the percentage of genes in each quadrant. The outer two rings represent the BCAS+cNGR-EVs group, and the inner two rings represent the BCAS+EVs group. An increased proportion of Q1 (red) and Q9 (blue) in the outer rings indicates a greater extent of transcriptomic preservation by cNGR-EVs compared with EVs across in inhibitory neurons, OPCs and astrocytes. Abbreviations: InN, inhibitory neurons; OPCs, oligodendrocyte progenitor cells; AC, astrocytes.
